# An ADAMTS Sol narae is required for cell survival in *Drosophila*

**DOI:** 10.1038/s41598-018-37557-9

**Published:** 2019-02-04

**Authors:** Orkhon Tsogtbaatar, Jong-Hoon Won, Go-Woon Kim, Jeong-Hoon Han, Young-Kyung Bae, Kyung-Ok Cho

**Affiliations:** 1 Department of Biological Sciences, Korea Advanced Institute of Science and Technology, 291 Daehak-ro, Yuseong-gu, Daejeon Korea; 2 Present Address: Center for Bioanalysis, Korea Research Institute of Standards and Science, 267 Gajung-ro, Yuseung-gu, Daejeon Korea

**Keywords:** Apoptosis, Developmental biology

## Abstract

Cell survival is essential for all living organisms to cope against multiple environmental insults. Intercellular signaling between dying and surviving cells plays an important role to ensure compensatory proliferation, preventing tissue loss after environmental stresses. Here, we show that Sol narae (Sona), a Disintegrin and metalloproteinase with thrombospondin motifs (ADAMTS) in *Drosophila* is required for cell survival. *sona* exhibited a positive genetic interaction with *Death-associated inhibitor of apoptosis 1 (Diap1*), and a negative genetic interaction with *reaper* (*rpr)*. Transcription patterns of *sona*, *Diap1*, and *rpr* genes in the pouch region of wing discs were coordinately changed after irradiation. Interestingly, there was a negative correlation in the expression levels of Sona and DIAP1, and both cell types, one with high Sona level and the other with high Diap1 level, were resistant to irradiation-induced cell death. The *sona*-expressing cells rarely entered into cell cycle themselves but promoted the nearby cells to proliferate in irradiation conditions. We found that these *sona*-expressing cells are able to upregulate Cyclin D (Cyc D) and increase tissue size. Furthermore, transient Sona overexpression increased survival rate and promoted development of flies in irradiation conditions. We propose that the two types of radiation-resistant cells, one with high Sona level and the other with high Diap1 level, communicate with dying cells and between each other for cell survival and proliferation in response to irradiation.

## Introduction

Multicellular organisms encounter stress conditions throughout their developmental and adult stages. Damaged cells by stresses and abnormal cell division need to be removed to ensure proper development and homeostasis. Apoptosis, programmed cell death, is an evolutionally conserved process for the removal of unwanted or damaged cells, which involves condensations of chromatins, shrinkage of cells, and activation of caspases that degrade cellular components^1–3^. Apoptosis accompanies compensatory proliferation to restore damaged tissues to the right size and shape^4,5^, for which intercellular communication between dying and surviving cells is essential^6–8^. The wing imaginal disc of *Drosophila melanogaster* is an excellent system to study apoptosis and compensatory proliferation, because wing discs exhibit little apoptosis during normal larval development but exhibit extensive apoptosis under stress conditions^9–11^. Wing discs with up to 40–60% of extensive cell death can yield normal adult wings, indicating that regeneration process in wing discs is efficient and robust^4,7,9,11–13^.

Diap1 is one of the most important proteins for cell survival under stress conditions. Diap1 is an E3 ubiquitin ligase that blocks cell death by tagging the caspases with ubiquitin for proteasome-mediated degradation^14,15^. Under severe stress conditions, the activity and the amount of Diap1 protein is decreased by the binding of pro-apoptotic proteins such as Head involution defective (Hid), Reaper (Rpr) and Grim^16–20^. Especially, binding of Hid stimulates autoubiquitination of Diap1 that results in degradation of Diap1^14,20,21^. Among these pro-apoptotic genes, *rpr* is expressed in a pattern most similar to that of dying cells^16^, and irradiation can activate transcription of *rpr* in dying cells through p53 binding to an enhancer of the *rpr* gene^22,23^. Heterozygous *Diap1* flies are more sensitive to damages than wild-type flies, demonstrating that the amount of Diap1 correlates with the extent of cell survival, and the cells enter the apoptotic process when the level of Diap1 falls below the critical point because of pro-apoptotic proteins^14,20,24^. Signaling pathways such as JAK-STAT and Hippo pathways are involved in controlling the transcriptional rate of Diap1^25–27^.

We recently reported that a *Drosophila* ADAMTS Sona is important for fly development and promotes Wg signaling^28^. Sona is processed to an active form in both intracellular and extracellular regions, and promotes Wg secretion. In general, ADAMTSs are secreted proteases that function in extracellular matrix (ECM). Six fly ADAMTSs are involved in various processes such as cell migration, organogenesis and cell signaling^29–31^. Similarly, nineteen mammalian ADAMTSs serve diverse roles^32^. Some are involved in processing ECM proteins, and malfunction of these ADAMTSs causes connective tissue disorder, arthritis, and arthrosclerosis. Other ADAMTSs regulate cell proliferation and cell survival, and their malfunction causes tumor development and metastasis. Despite involvement of ADAMTSs in diverse cellular functions, the underlying mechanisms of these ADAMTSs are still largely unknown.

We report here that *sona* is required for cell survival. *sona* is expressed in a patchy pattern in the wing disc, and irradiation coordinately changed transcription of both *sona* and *Diap1* with negative correlation. Cells expressing either *sona* or *Diap1* at a high level did not exhibit cell death, indicating these two types of cells are resistant to cell death. Consistent with their response to irradiation, *sona* exhibited a positive genetic relationship with *Diap1* but negative genetic relationship with *rpr*. Furthermore, Sona upregulated the level of Cyc D and increased cell proliferation in a cell non-autonomous manner. We propose that the intercellular signaling between the two types of surviving cells, one expressing *sona* and the other expressing *Diap1*, plays an important role for cell survival and compensatory proliferation.

## Results

### Loss of *sona* results in cell death

**Figure 1:**
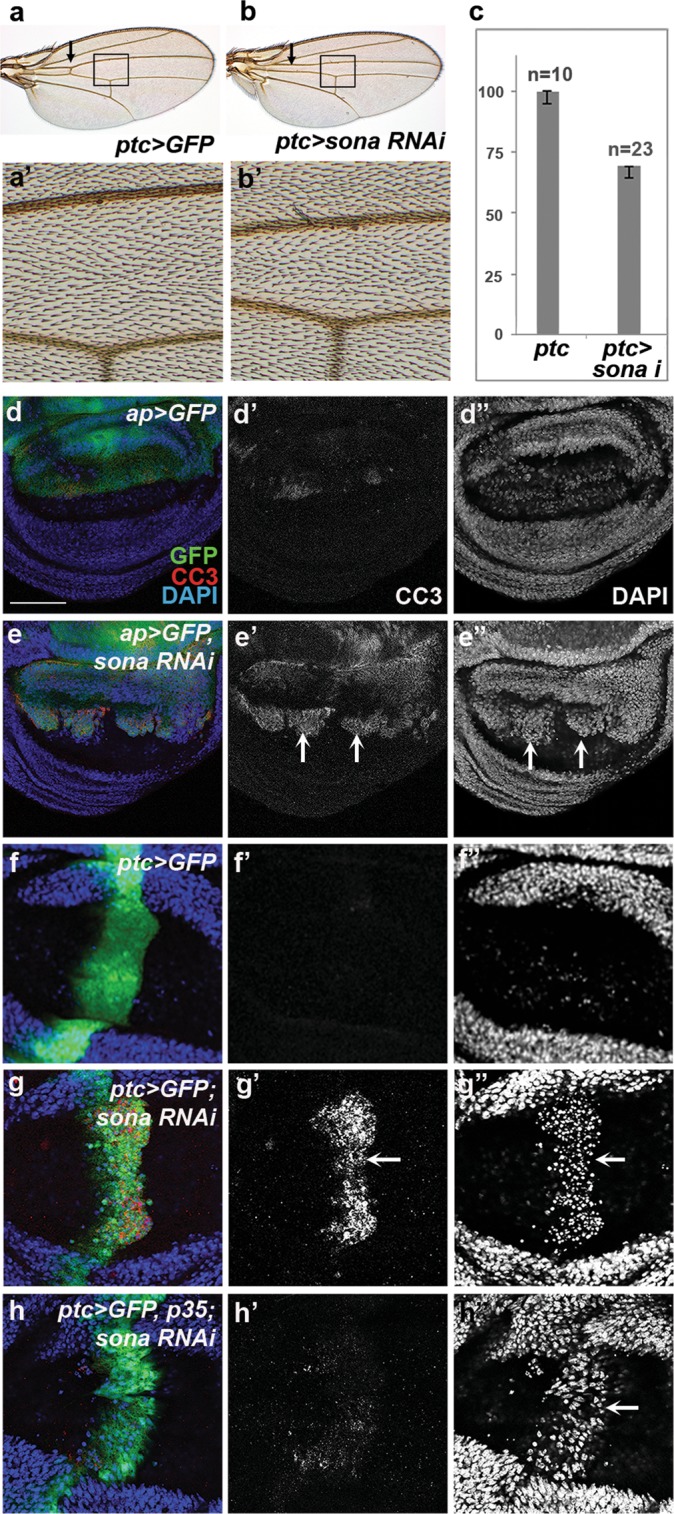
Loss of *sona* causes apoptosis. Genotypes of wing discs and the visualized proteins are indicated at the upper and lower right of confocal images in all figures, respectively. (**a–c**) control *ptc* > *GFP* (**a**) and *ptc* > *sona RNAi-1*^*11–4*^ (**b**) wings cultured at 18 °C. Arrows in (**a**,**b**) indicate presence and absence of anterior cross-veins, respectively. The regions marked with the black boxes in (**a**,**b**) are magnified in **a’** and **b’**. (**c**) The distance between L3 and L4 veins in **a’** and **b’** were measured and graphed. Sample numbers are indicated at the top of bars. (**d**,**e**) Dorsal cells with CC3 and nuclei are marked with arrows in **e’** and **e”**. (**f–h**) CC3 signals and pyknotic nuclei at the basal region are marked with arrows. Scale bars: (**d**,**e**) 60 μm; (**f–h**) 40 μm.

We previously reported that expression of *sona RNAi* driven by various *Gal4* lines results in lethality and malformed appendages^28^. *sona-RNAi-1* and *-2* lines were generated by using two different regions of the *sona* cDNA, and these RNAi lines driven by various *Gal4* lines exhibit same phenotypes but with varied strengths^28^. For instance, *engrailed (en)-Gal4* > *sona RNAi-1*^*3–23*^ *(en* > *sona RNAi-1*^*3–23*^*)* wings were smaller in the posterior region (Supplementary Fig. S1a,b). The average distance between L3 and L4 veins was only about 70% of the control (n = 10), and anterior cross-vein was absent in 40% of *patched (ptc*) > *sona RNAi-1*^*11–4*^ wings cultured at 18 °C (n = 23) (Fig. 1a–c). Hair density in the L3-L4 region, however, was unchanged (Fig. 1a,b). Thus, the loss of *sona* caused reduction in cell number but not cell size.

We then examined whether cell death is responsible for the reduced cell number in *sona RNAi-*expressing wings. Indeed, *sona RNAi* expressed by *apterous (ap)-*, *ptc-*, and *en-Gal4s* increased cell death detected by an antibody generated against the cleaved form of human Caspase 3 (CC3) that indicates fly Dronc activity (Fig. 1e,g; Supplementary Fig. S1c)^33–35^. The affected dorsal domain in *ap* > *sona RNAi-1*^*11–4*^ discs exhibited a high level of CC3, and highly condensed nuclei were present in the basal region (Fig. 1d,e; Supplementary Fig. S1d,e). Highly condensed nuclei were also present in the anterior-posterior boundary of *ptc* > *sona RNAi-1*^*11–4*^ discs (Fig. 1f,g). When p35 that inhibits the activity of effector caspases^36^ was coexpressed with *sona RNAi*, the level of CC3 was reduced and the morphology of nuclei became normal although the nuclei still remained at the basal region (Fig. 1h). Therefore, p35 largely rescued the loss of *sona* phenotypes.

### Different kinds of *sona* clones are generated

**Figure 2:**
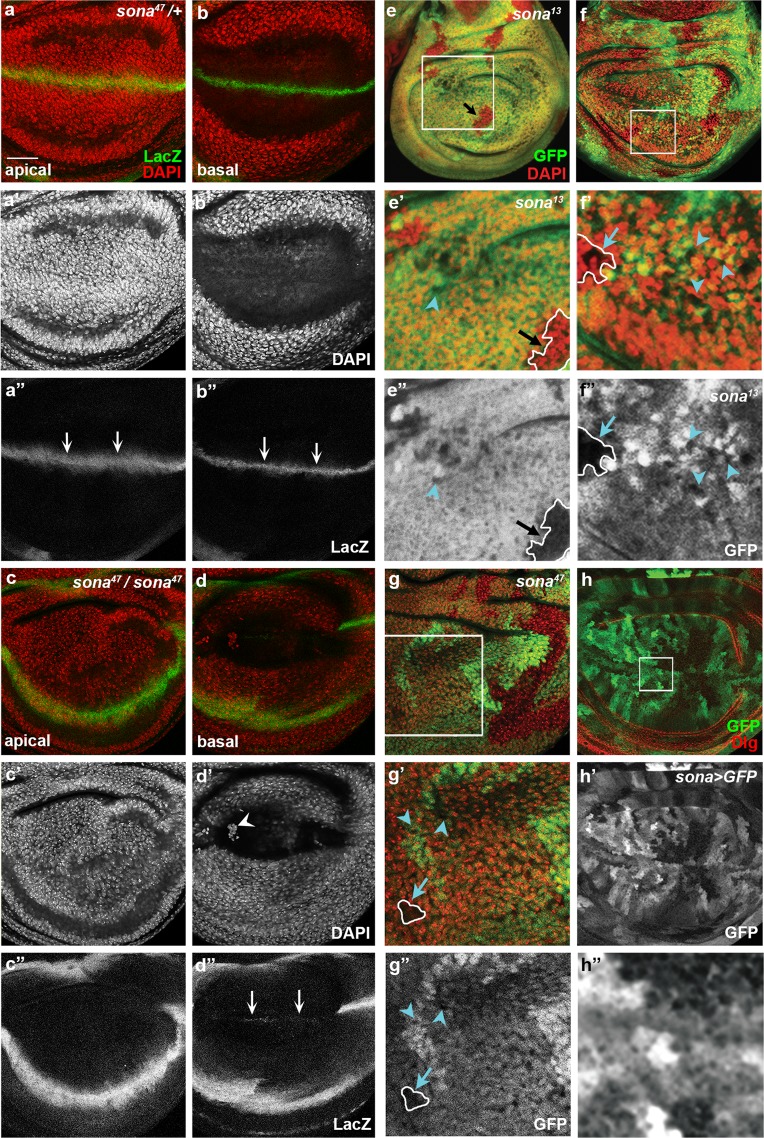
Mosaic pattern of *sona* expression may be responsible for the three types of *sona* clones. Confocal planes are indicated at the lower left. (**a–d**) The effects of *sona*^*47*^ mutation on the survival of Wg-lacZ cells and the number of nuclei. Wing discs of heterozygous (**a**,**b**) and homozygous *sona*^*47*^ allele (**c**,**d**) are used. The loss of *wg-lacZ* cells is marked by arrows at the apical region in **a”** compared to **c”** and at the basal region in **d”** compared to **b”**. Large, medium-sized, and small *sona*^*13*^ and *sona*^*47*^ clones are marked with black arrows, blue arrows, and blue arrowheads, respectively. The regions marked with white boxes in **e–g** are magnified in **e’–g’**. (**h**) Expression pattern of GFP in *sona* > *GFP* wing discs. The region in the white box (**h**) is magnified in **h”**. Scale bars: (**a**–**d**,**g**,**h**) 40 μm; (**e**,**f**) 60 μm; (**e’**) 23.7 μm; (**f’**) 14.5 μm; (**g’**) 21 μm; (**h”**) 7.2 μm.

To further prove that Sona functions in cell survival, we examined whether *sona*^*47*^ wing discs show any sign of cell death. Because Sona is involved in Wg signaling, we wanted to check whether the role of *sona* in cell survival is specific to Wg-producing cells or general to cells in the pouch. To this end, we recombined *sona*^*47*^ allele with the wg-LacZ marker *wg*^*en-11*^^37^, and found that the wing pouch region of homozygous *sona*^*47*^ discs had less number of nuclei compared to that of heterozygous *sona*^*47*^*/*+ discs at the apical region (compare Fig. 2a,c’). At the basal region, condensed nuclei were detected in the *sona*^*47*^ discs but not in *sona*^*47*^*/*+ discs (compare Fig. 2b’,d’). Furthermore, Wg-LacZ expression was absent in the DV midline at the apical region and barely visible at the basal region of *sona*^*47*^ discs (compare Fig. 2a” and c”, arrows in b” and d”). These results showed that *sona* is important for the survival of both wing pouch cells and *wg-lacZ* cells.

We then examined *sona*^*13*^ and *sona*^*47*^ clones marked by the absence of GFP in discs. Interestingly, the size and cell density of *sona* clones were widely varied (Fig. 2e–g). Based on the difference in clone size, we arbitrarily assigned the clones to three groups. The first group represented single-cell clones with no nuclei, which may absolutely require Sona (blue arrowheads in Fig. 2e–g). The second group of clones was larger than the first group of clones and their nuclei were sparser than their heterozygous counterparts (blue arrows in Fig. 2f,g). Thus, these cells may require Sona at a lesser extent than the cells in the first group of clones. The third group of clones was large in size and the cell density was same as heterozygous counterparts (black arrows in Fig. 2e–e”). Thus, the cells in these clones did not require Sona.

These different *sona* clones reminded us of the patchy pattern of GFP driven by the *sona-Gal4* line, P{GawB}CG9850. The intensity of GFP in the *sona* > *GFP* disc varied widely in a highly magnified image (Fig. 2h), and has been shown to be similar to the *in situ* hybridization pattern of *sona* transcripts^28^. To further prove this point, we co-stained *sona* > *GFP* discs for GFP and Sona protein with anti-GFP and Sona-Pro antibody^28^. We found that the level of GFP correlated with that of Sona protein in *sona* > *GFP* wing discs, indicating that GFP driven by *sona-Gal4* driver reflects both *sona* transcript and Sona protein (Supplementary Fig. S2). Therefore, the three different kinds of *sona* clones may have been generated by the differences in the level of *sona* expression.

We expected to see the *sona* clones in the entire pouch region because *sona* was expressed in the entire wing pouch region. Indeed, the large *sona*^*13*^ clones were generated randomly without any preferred location (Supplementary Fig. S3). Overall shapes of large *sona* clones were similar to those wedge-typed control clones but *sona* clones appeared to be somewhat rounder than the wild-type clones. To further identify differences between wild-type clones and *sona* clones, we next generated *sona RNAi* clones (Supplementary Fig. S4). We collected embryos from *hsFlp* > *Actin* > *CD2* > *Gal4 UAS-GFP* and *hsFlp* > *Actin* > *CD2* > *Gal4 UAS-GFP UAS-sona RNAi-1*^*11–4*^ flies for one day at room temperature, and heat-shocked them for one hour at 37 °C at five different time points (Supplementary Fig. S4a). Examination of GFP-positive wild-type clones and *sona* RNAi clones revealed that average area of RNAi clones are considerably less than that of wild-type clones (Supplementary Fig. S4b–j). When the clones were generated at the 3^rd^ instar stage that was 29 hours before dissection, wild-type clones were evenly distributed in the wing pouch but *sona* RNAi clones were absent in some particular regions (Supplementary Fig. S4k–m). These data are consistent with our finding that cells in the pouch region differentially require Sona.

### The extent of cell survival is proportional to the level of *sona* expression in irradiated conditions

**Figure 3:**
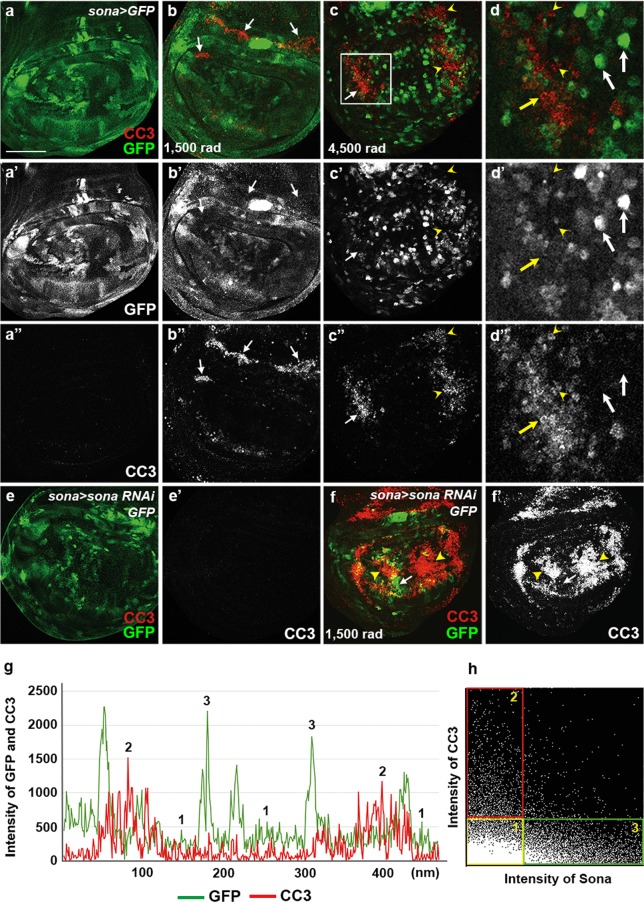
Sona-expressing cells are resistant to cell death upon irradiation. Irradiation intensity is indicated at the lower left. (**a–d**) Control *sona* > *GFP* wing disc (**a**), *sona* > *GFP* wing discs from the larvae irradiated at 1,500 rad (**b**) and at 4,500 rad (**c**) followed by culture for 24 hrs before dissection. The white box in **c** is magnified in **d**. The yellow arrows indicate the cells that have a high level of CC3 signals and a low level of *sona*. The arrowheads mark the cells with no *sona* but with CC3. The white arrows mark the cells with a high level of *sona* without CC3. (**e**,**f**) Control (**e**) and irradiated (**f**) *sona* > *sona RNAi-1*^*11–4*^ wing disc from the irradiated larvae that were cultured for 24 hours before dissection. Arrows and arrowheads mark the regions with GFP and CC3 signals, respectively. (**g**,**h**) The chart displays relationship between *sona* (GFP) and CC3 levels as described in Supplementary Fig. 2a (**g**). A 2D histogram showing the inverse correlation between *sona* (LacZ) and CC3 values, and the three arbitrary groups of cells are indicated by colored boxes (**h**). Representatives of these three groups are marked in **g**. Scale bars: (**a**) 60 μm; (**b**,**c**,**e**,**f**) 40 μm; (**d**) 13.2 μm.

To understand the effect of *sona* level on cell survival, we examined which cells are more prone to cell death under γ-ray irradiation. Based on our clonal analyses, we expected that the cells expressing *sona* at the high level should be more resistant to cell death. To test this idea, *sona* > *GFP* larvae were irradiated with γ-ray at 1,500 or 4,500 rad, and the pattern of GFP and CC3 in their wing discs were examined (Fig. 3a–d). Indeed, CC3 signals were mostly absent in the cells that express a high level of *sona* (Fig. 3d). Cell death by irradiation was severer in *sona* > *sona RNAi-1*^*11–4*^; *GFP* discs than control *sona* > *GFP* discs, demonstrating that the reduction in *sona* expression enhances cell death (compare Fig. 3b,e to f). Thus, the transcriptional level of *sona* seemed to positively correlate with the extent of cell survival under the irradiation condition.

To quantify the relationship between the levels of *sona* and cell death, lines were randomly drawn and the intensities of GFP and CC3 on those lines were measured in irradiated *sona* > *GFP* discs (Supplementary Fig. S5a). Consistent with the immunocytochemical data in Fig. 3c, raw data showed that the level of CC3 was low when the level of GFP was high, and vice versa in irradiated discs (16 measurements in 10 discs) (Fig. 3g). To statistically analyze these data, we obtained the Mander’s coefficient using measured intensities from multiple wing discs. The Mander’s coefficient is a widely used statistical method that quantifies the degree of co-localization between two fluorescent channels by the combined measurement of correlation and co-occurrence (values ranging from 0 to 1; 0 for complete mutual exclusion, 0.5 for neutral relation, and 1 for complete co-occurrence)^38^. The average Mander’s coefficient was 0.13 for Sona^+^ when CC3 is present and 0.15 for CC3^+^ when Sona is present (Supplementary Fig. S5b,c), which indicate their strong negative correlation. The analysis with the irradiated *en* > *GFP* was carried out as a control to validate the use of Mander’s coefficient for correlation analysis (Supplementary Fig. S6a,b). In sum, Mander’s coefficient values confirmed that Sona and CC3 levels show an inverse correlation after irradiation.

Intensity values of GFP and CC3 in *sona* > *GFP* and *en* > *GFP* discs were used to generate 2D intensity histograms in order to obtain quantitative relationship. Intensity values of different sample sources generated similar 2D histograms, and representatives are shown in Fig. 3h and Supplementary Fig. S6c. We also created an artificial case that represents perfect positive correlation between the two values and generated 2D histogram for comparison (Supplementary Fig. S6d,e). Unlike these two controls, the 2D histogram of the *sona* > *GFP* discs showed inverse correlation between GFP and CC3 as expected from the data of Mander’s coefficient (Fig. 3h). We arbitrarily categorized the values in the 2D histogram into three groups as follows. Cells with the low levels of both Sona and CC3 were assigned to group 1 (yellow box), cells with the low level of Sona and the high level of CC3 were assigned to the group 2 (red box), and cells with the higher level of Sona and the low level of CC3 were assigned to the group 3 (green box). Cells in the groups 2 and 3 were readily identified in the wing discs and were consistent with our finding that the level of *sona* determines cell survival (Fig. 3d). On the contrary, the 2D histogram clearly identified the group 1 cells that represented the majority of cells in the wing disc, but were undetectable in the stained discs. These cells had no *sona* or a very low level of *sona* expression but were resistant to cell death. We reasoned that the group 1 cells may express another protein that inhibits cell death, and we chose Diap1 as a candidate because Diap1 is widely expressed in wing discs and essential for cell survival in a cell autonomous manner^19^.

### Expression patterns of *sona*, *Diap1*, and *rpr* show negative correlation after irradiation

**Figure 4:**
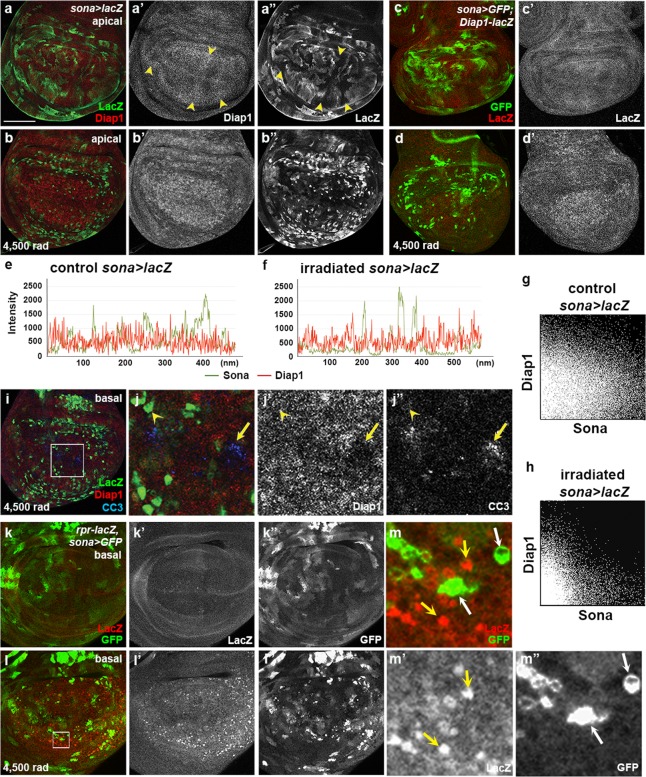
Negative correlation of *sona* and *diap1* patterns is accentuated by irradiation. (**a**,**b**) Patterns of Diap1 and LacZ in control (**a**) and *sona* > *lacZ* wing discs irradiated at 4,500 rad (**b**). Arrowheads mark the region with negative correlation, a high level of Diap1 and a low level of LacZ (**a’**,**a”**). (**c**,**d**) Patterns of *Diap1-lacZ* and GFP in control (**c c’**) and irradiated *sona* > *GFP; Diap1-lacZ* wing discs at 4,500 rad (**d**,**d’**). (**e**,**f**) The charts display the expression level between *sona* (LacZ) and Diap1 in control (**e**) and irradiated *sona* > *lacZ* wing discs at 4,500 rad (**f**). (**g**,**h**) The 2D histograms show correlation between *sona* (LacZ) and Diap1 values in the discs before (**g**) and after irradiation (**h**). (**i**,**j**) Basal plane of the irradiated *sona* > *lacZ* disc. The white box in **i** is magnified in **j**. Arrow mark CC3 signals in the cell with no Diap1, and arrowheads mark the cell with *sona* but no CC3 signals. (**k–m**) The pattern and level of *rpr-LacZ* and *sona* (GFP) in control (**k**) and in irradiated (**l**) in *rpr-lacZ*, *sona* > *GFP* discs. The region marked with a white box in **l** is magnified in **m**. Cells with no GFP, but with a high level of LacZ are marked with yellow arrows, and cells with a high level of GFP, but with no LacZ are marked with white arrows. Scale bars: (**a**–**d**,**i**) 60 μm; (**j**) 15.4 μm; (**k**,**l**) 40 μm; (**m**) 5.7 μm.

To find out the relationship between *sona* and Diap1, we examined the expression patterns of Diap1 and LacZ in *sona* > *lacZ* discs (Fig. 4a). LacZ and Diap1 exhibited complicated mosaic patterns with weak negative correlation (arrowheads in Fig. 4a’,a”). When the *sona* > *lacZ* larvae were irradiated at 4,500 rad, LacZ pattern was changed to cluster-like and Diap1 was more evenly expressed in the wing pouch (Fig. 4a,b). Raw data and 2D intensity histograms of *sona* > *GFP* control and irradiated discs revealed that the level of Diap1 was low in the LacZ^+^ clusters (Fig. 4e–h; Supplementary Fig. S7). This indicated that negative correlation between *sona* and Diap1 became more strengthened by irradiation. LacZ pattern in *sona* > *GFP*; *Diap1-LacZ* discs was similar to the pattern of Diap1 protein in *sona* > *lacZ* discs, demonstrating that the negative correlation between *sona* and *Diap1* is generated at the transcriptional level (Fig. 4c,d). Thus, the transcription of both *sona* and *Diap1* genes are coordinately changed by irradiation with negative correlation.

Since both Sona and Diap1 are involved in cell survival and their expressions are negatively correlated, we expected that the cells with neither Sona nor Diap1 become apoptotic upon irradiation. Indeed, cells with the high level of CC3 had low levels of *sona* and *Diap1* expression, which may belong to the group 2 cells that had CC3 signals and a low level of *sona* (Fig. 4i,j; Fig. 3h). Taken together, expression of either *sona* or *Diap1* at a high level is sufficient for cell survival against irradiation.

If *sona* is expressed at a high level in surviving cells, *sona* and *rpr* should also be expressed with the negative correlation because *rpr* is expressed in dying cells. Consistent with previous reports^22^, irradiation changed Rpr-LacZ pattern and increased the level of LacZ in *rpr-lacZ/sona-Gal4*; *UAS-GFP/*+ wing discs (Fig. 4k,l). Furthermore, *rpr* was expressed at a high level in the cells with no *sona* expression (yellow arrows in Fig. 4m,m’). Conversely, cells with a high level of *sona* expressed no *rpr* (white arrows in Fig. 4m,m’). In summary, *rpr* is upregulated in the cells with no *sona* expression in irradiation conditions.

### *Sona* exhibits genetic relationships with *Diap1* and *rpr*

**Figure 5:**
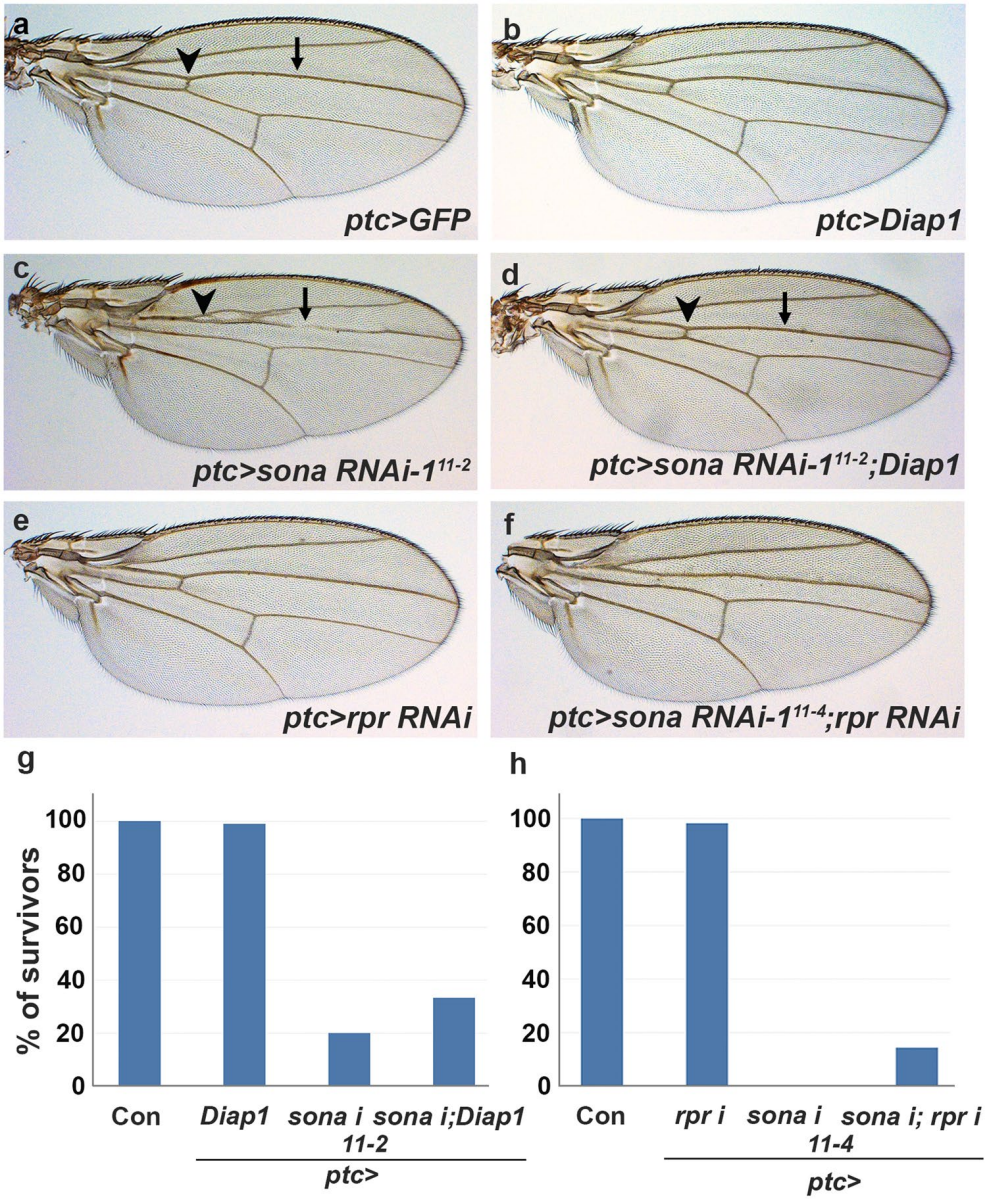
*sona* exhibits genetic relationship with *diap1* and *rpr*. Genotypes are indicated at the lower right. Arrowheads and arrows indicate the anterior crossvein and the L3 vein, respectively. (**a–f**) Adult wing phenotype by single or co-expression of *sona RNAi*, *rpr RNAi*, and Diap1 by *ptc-Gal4* driver cultured at 25 °C. *ptc* > *sona RNAi-1*^*11–2*^; *Diap1* wings (**d**) have anterior cross-vein (arrowhead) and normal L3 vein (arrow). *ptc* > *sona RNAi-1*^*11–4*^ flies show pupal lethality (**h**), while *ptc* > *rpr RNAi*; *sona RNAi-1*^*11–4*^ flies survive to adulthood, and still show reduction in distance between L3 and L4 veins (**f**). (**g**,**h**) Bar graphs show the effects of *diap1* and *rpr RNAi* expression on *sona RNAi* phenotype.

Our data so far have shown that *sona* and *Diap1* are expressed in different cells but function together for cell survival. To examine their genetic relationship in cell survival, we compared the wing phenotypes of *sona RNAi*, *Diap1* or both, driven by *ptc-Gal4*. While all *ptc* > *Diap1* wings were normal, 83% of *ptc* > *sona RNAi-1*^*11–2*^ (n = 85) were abnormal with no anterior cross-vein, partially lost L3 vein, and narrowed intervein region between L3 and L4 veins (Fig. 5a–c). When both *Diap1* and *sona RNAi-1*^*11–2*^ were co-expressed, however, only 10% (n = 97) were abnormal (Fig. 5d). Thus, the wing phenotype of *ptc* > *sona RNAi-1*^*11–2*^ flies was considerably rescued by co-expression of *Diap1*. In addition, while only 20% (n = 85) of *ptc* > *sona RNAi-1*^*11–2*^ flies reached adult stage, 33% (n = 97) of *ptc* > *sona RNAi-1*^*11–2*^; *Diap1* flies could reach adult stage (Fig. 5g). Thus, *Diap1* partially suppressed the lethality caused by the knockdown of *sona*. This suggests that *sona* and *Diap1* exhibit a positive genetic relationship.

We next tested whether *sona* has any genetic relationship with *rpr*. *ptc* > *rpr RNAi* flies had normal wing phenotype with no lethality, and *ptc* > *sona RNAi-1*^*11–4*^ flies cultured at 25 °C exhibited 100% of pupal lethality (Fig. 5e,h). When both *rpr RNAi* and *sona RNAi-1*^*11–4*^ were coexpressed, up to 14% of *ptc* > *rpr RNAi*; *sona RNAi-1*^*11–4*^ flies (n = 63) reached adult stage with somewhat defective wings (Fig. 5f,h). This indicated that cell death by loss of *sona* partially depends on the function of *rpr*. Taken together, *sona* exhibited the positive genetic relationship with *Diap1* but the negative genetic relationship with *rpr*, which is consistent with our finding that *sona* promotes cell survival.

### *sona*^+^ clusters are formed by increased *sona* transcription but not by cell division

We have shown that irradiation changes the pattern of *sona-Gal4* expression (Figs 3c, 4b). The most noticeable change in irradiated *sona* > *GFP* discs was the appearance of *sona*^+^ clusters. *sona*^+^ clusters were generated by 4,500 rad but rarely by 1,500 rad (Fig. 3a’–c’). As a result, mostly two major types of cells remained in the wing pouch after irradiation at 4,500 rad: one with the high level of *sona* and the other with no or very low *sona* expression (Fig. 3c; Fig. 4b,d,i,l). *en* > *GFP* or *tubulin* > *GFP* larvae irradiated at 4,500 rad did not show any change in the pattern of GFP in the wing discs, so the GFP-positive clusters in *sona* > *GFP* discs were unique to *sona*-expressing cells (Supplementary Fig. S6a, S8).

**Figure 6:**
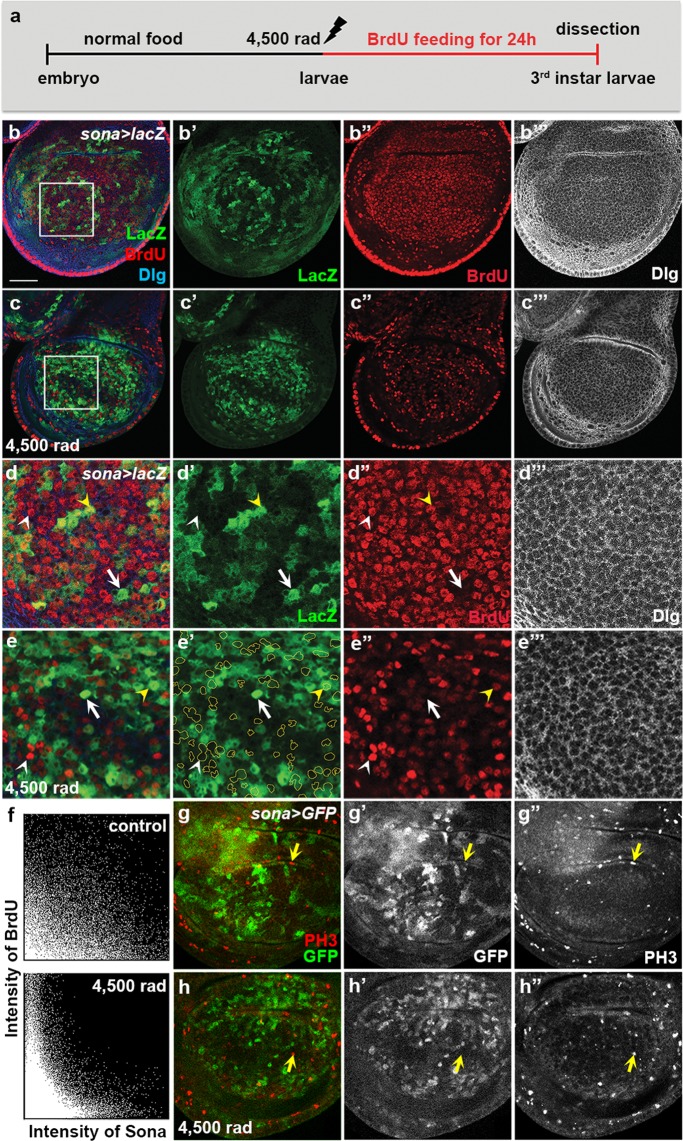
Cells expressing a high level of Sona do not divide upon irradiation. (**a**) The experimental scheme to examine the effect of irradiation on *sona* expression and DNA synthesis. (**b–e**) Wing discs from the control (**b**) and irradiated (**c**) *sona* > *lacZ* larvae fed with BrdU for 24 hours before dissection. The boxed regions in (**b**,**c**) are magnified in (**d**,**e**), respectively. The cells expressing a high level of *sona* are outlined to show the lack of overlapping between LacZ and BrdU signals in **e’**. An arrow marks a representative *sona*-expressing cell lacking BrdU incorporation while white and yellow arrowheads mark incorporation of BrdU in the cells with no or a low level of *sona* expression, respectively. (**f**) 2D histograms showing the inverse relationship between the levels of BrdU and *sona* in control and irradiated *sona* > *lacZ* discs above. (**g**,**h**) Phospho-histone H3 patterns of control (**g**) and irradiated (**h**) *sona* > *GFP* discs. Arrows mark the cells with PH3 but no GFP. Scale bars: (**b**,**c**,**g**,**h**) 40 μm; (**d**,**e**) 12.6 μm.

Based on the result that *sona*^+^ clusters are formed by irradiation, these *sona*^+^ clusters may be generated by cell division or induction of *sona* transcription. To distinguish these possibilities, *sona* > *lacZ* larvae were irradiated at 4,500 rad, and then cultured in Bromodeoxyuridine (BrdU)-containing food for 24 hours until dissection (Fig. 6a). In control discs, BrdU was incorporated in the cells of the wing pouch region regardless of *sona* expression (Fig. 6b,d). In contrast, most *sona*-expressing cells did not incorporate BrdU but the neighboring cells did in irradiated discs (Fig. 6c,e). The two 2D intensity histograms also confirmed that *sona*-expressing cells have less BrdU incorporation (Fig. 6f). The histogram also revealed that a weak inverse correlation exists between *sona* expression and BrdU incorporation even in control discs (Fig. 6f). Therefore, *sona*-expressing cells divide less frequently than *sona*^−^ cells even in the unirradiated condition. Consistent with the BrdU data, mitotic cells marked with anti-phospho-histone H3 (PH3) antibody were mostly absent in the *sona*-expressing cells in both control and irradiated wing discs of *sona* > *GFP* larvae (Fig. 6g,h). We concluded that *sona*^+^ clusters are formed by increased *sona* transcription but not by cell division.

To further understand how these *sona*^+^ clusters are formed, we generated 3D and combined images from multiple confocal images of unirradiated and irradiated *sona* > *GFP* discs (Supplementary Fig. S9). Unlike the single confocal images and the combined images (Supplementary Fig. S9a–a”,b–b”), the 3D images revealed that the *sona*^+^ clusters were actually two dimensional confocal images from a continuous cell group that express *sona* at a high level (arrows in Supplementary Fig. S9c’). An image tilted from the 3D image showed that these continuous cell groups may be clonal (Supplementary Fig. S9d). When the combined images of *sona* expression were compared between irradiated and unirradiated discs, it appeared that irradiation induced either increase or decrease in the expression level of *sona*, leading to the greater difference between *sona*^+^ and *sona*^−^ cells (Supplementary Fig. S9a”,b”).

### Sona promotes development and survival under the irradiation condition

Generation of *sona*^+^ cell groups suggested that increase in *sona* transcription may be required for cell survival in the irradiation condition. To test this, we dissected *sona* > *GFP* wing discs at different time points after irradiation. We found that the level of GFP was same in both unirradiated and irradiated discs initially but was about doubly increased by 24 hours after irradiation (n > 30, Supplementary Fig. S10). Furthermore, the level of Sona protein was increased where GFP level is high in irradiated discs (Supplementary Fig. S11). These data raised an interesting possibility that increase in *sona* transcription is an important required step for survival against irradiation.

To test whether the increase in *sona* level can promote survival, we examined the effect of overexpressed Sona on irradiated flies. We have previously reported that overexpression of Sona by most *Gal4* drivers causes organismal death^28^. To overcome this Sona-induced lethality, we cultured *sona-Gal4/*+; *Gal80*^*ts*^/+ control flies and *sona-Gal4/*+; *Gal80*^*ts*^ *UAS-sona/*+ experimental flies at 18 °C for 6 days until the mid to late third instar stage, and then cultured them at 29 °C for six hours in order to transiently induce Sona expression. Flies were then irradiated with 3,000 rad or 4,500 rad, and cultured at 18 °C until adult stage. Control groups that were treated the same way without irradiation developed at the same pace regardless of Sona, and developed faster than irradiated flies. Interestingly, *sona-Gal4/*+; *Gal80*^*ts*^ *UAS-sona/*+ flies developed faster than the irradiated control flies (Supplementary Fig. S12). Furthermore, they produced more adults (20.5%, 15 adults out of 74 pupae) than control *sona-Gal4/*+; *Gal80*^*ts*^/+ flies (13.6%, 8 adults from 59 pupae). Although, Sona was overexpressed only for 6 hours prior to irradiation, both survival and development of flies were noticeably promoted.

### *sona*^+^ cells induce the expression of Cyc D in neighboring cells

**Figure 7:**
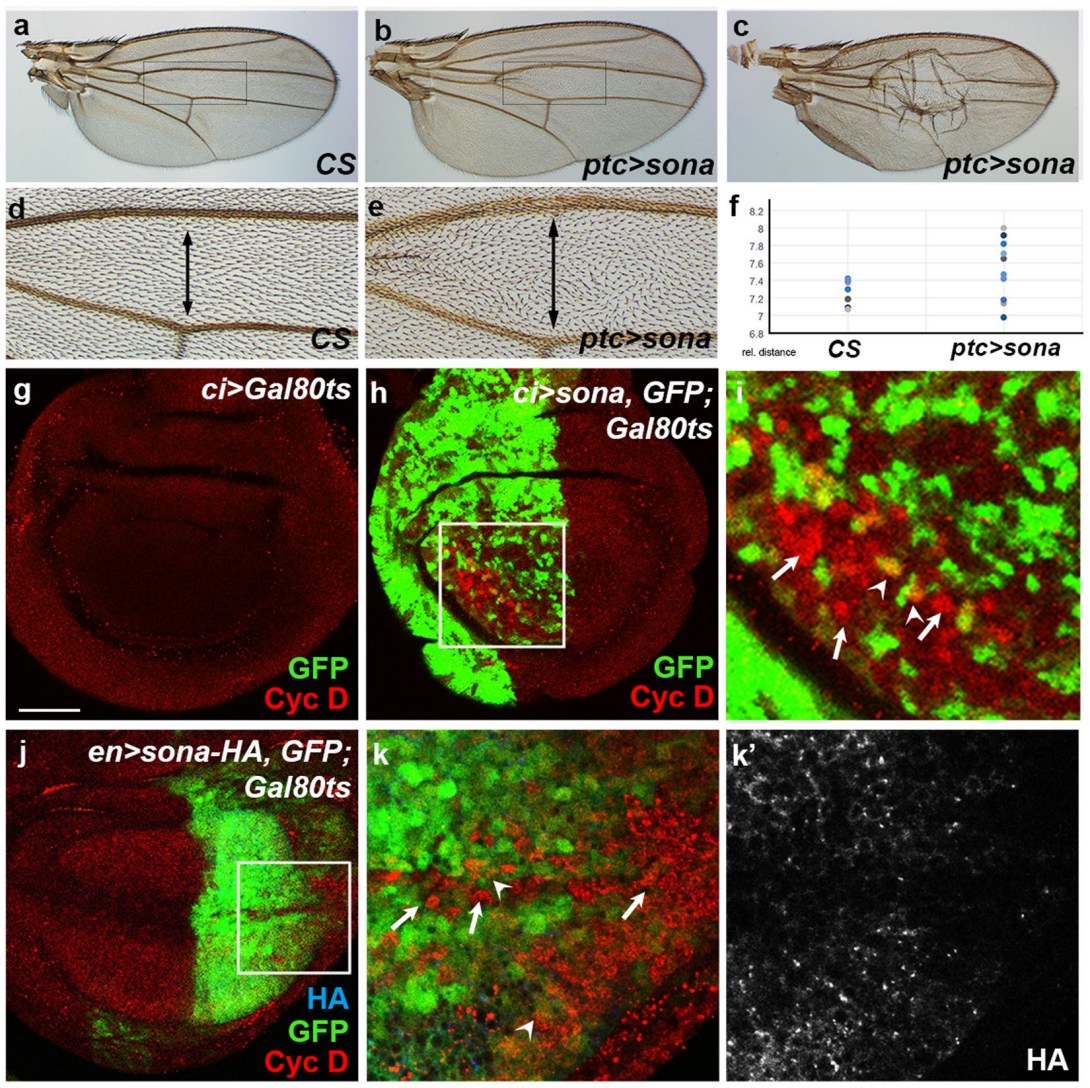
Sona overexpression induces tissue growth and the expression of Cyc D in neighboring cells. (**a**) Control *CS* adult wing. (**b**,**c**) The overgrowth phenotypes of two *ptc* > *sona* adult wings. The phenotype of **c** seems more severe than **b**. The boxed regions in (**a**,**b**) are magnified in (**d**,**e**), respectively. The lines with double arrowheads mark the region between L3 and L4 veins. (**f**) The quantitative analysis on the distances between L3-L4 regions in *CS* and *ptc* > *sona* wings. (**g–i**) Control *ci* > *Gal80*^*ts*^ (**g**) and *ci* > *sona*, *GFP*, *Gal80*^*ts*^ (**h**) larvae were cultured at 30 °C for 10 hours to induce transient overexpression of Sona and then their wing discs were stained for Cyc D. The boxed region in (**h**) is magnified in (**i**). Similar results in another disc are shown in Supplementary Fig. S13b. (**j**,**k**) *en* > *sona-HA*, *GFP*, *Gal80*^*ts*^ larvae were also used to induce Cyc D expression by Sona-HA. Larvae were cultured at 30 °C for 24 hours and stained for HA and Cyc D. Arrows mark the *sona*^−^ cells expressing Cyc D. Arrowheads mark the cells with upregulated Cyc D in the cells that are in the close vicinity of *sona*^+^ cells. Scale bars: (**g**,**h**) 40 μm; (**i**,**k**) 17 μm; (**j**) 50 μm.

As the neighboring cells of *sona*^+^ clusters incorporated BrdU, and transient expression of Sona increased survival rate, we hypothesized that the *sona*^+^ cells send signals to neighboring cells for cell proliferation and enhance survival. To test whether Sona is able to promote cell proliferation and increases tissue size, we overexpressed Sona using *ptc-Gal4* driver at 18 °C. Most *ptc* > *sona* flies were lethal, but wings of rare survivors (~70%, n = 16) exhibited enlarged *ptc* region (Fig. 7a–e). Some wings had enlarged bubble in the *ptc* region, which supports that Sona is able to increase tissue size. The distances between the L3 and L4 veins of these wings except the one with bubbles are plotted in Fig. 7f.

We then examined how Sona is able to increase tissue size. Since prolonged overexpression of *sona* causes lethality^28^, we used *Gal80*^*ts*^^39^ to transiently induce *sona* in the anterior region by *cubitus interruptus (ci)-Gal4* driver. *UAS-GFP*; *UAS-sona*, *Gal80*^*ts*^ flies were crossed with *c*i*-Gal4* flies and cultured at 18 °C, and the progeny was shifted to 29 °C for 6, 10, and 14 hours during the third instar stage to transiently induce Sona. Because Sona can increase tissue size, we examined whether the level of any Cyclins is increased in the wing discs. Six hour culture at 29 °C did not induce GFP at all, and fourteen hour culture induced signs of tissue damage, so we examined the wing discs after 10 hour induction (data not shown). Among the three larval progeny, *ci* > *Gal80*^*ts*^ larvae and *ci* > *GFP*; *Gal80*^*ts*^ larvae did not show any change (Fig. 7g; Supplementary Fig. 13a) but *ci* > *GFP*; *sona*, *Gal80*^*ts*^ larvae had upregulated Cyc D in wing discs (Fig. 7h; Supplementary Fig. 13b). Cyc D was expressed in the cells that are in the close vicinity of but not in *sona*-expressing cells in most cases (Fig. 7i). Similar experiments were carried out in the posterior region with *en-Gal4* driver, in which GFP was detected after 24 hours of transient expression (Fig. 7j,k). We used *UAS-sona-HA* instead of *UAS-sona* for transient expression in order to check the expression of ectopic Sona with anti-HA antibody (Fig. 7k’). Similar to the experiment done in the anterior region with *ci-Gal4*, Cyc D was upregulated in the cells that show neither GFP nor HA in the posterior region with *en-Gal4* (Fig. 7k). Cyc D is a key Cyclin that responds to mitogens before the initiation of DNA synthesis, and its expression is regulated by Wnts in mammalian system^40–43^. These data strongly suggest that Sona-producing cells send out signals to the neighboring cells for induction of Cyc D. Whether the signal is Sona itself, Wg, or unknown molecules needs further analysis.

## Discussion

We have shown in this report that Sona functions in two different ways. First, Sona is required cell autonomously for cell survival, and the level of *sona* transcription correlates with the degree of cell survival under the irradiated condition. We found that cells expressing a high level of either *sona* or *Diap1* are resistant to cell death, so there are at least two different types of surviving cells in wing discs. Second, Sona induces cell proliferation cell non-autonomously. Transient expression of *sona* upregulated Cyc D in neighboring cells but not in *sona-*expressing cells, suggesting that radiation-resistant *sona-*expressing cells secrete signaling molecule(s) that upregulates Cyc D in neighboring cells. In conjunction with genetic interactions between *sona*, *Diap1* and *rpr*, cells expressing *sona* may dynamically interplay with other cells that express *Diap1* and *rpr* for damage control.

Sona is expressed in the patchy pattern, and *sona* clones with different sizes were generated in the wing pouch region (Fig. 2). We speculate that the large *sona* clones are generated where *sona* is not expressed. Similar result was reported in the loss-of-function clone analysis of the *vestigial* (*vg*) gene that is essential for survival and proliferation of wing disc cells^43^. The authors found that *vg RNAi* clones are generated where Vg is not expressed or expressed at a low level in wing discs^44^. That is, the loss-of-function *vg* clones are rarely formed where Vg is required for cell survival and proliferation. Indeed, Vg drives cell cycle progression by inducing *dE2F1* gene whose product is essential for G1 to S transition in cell cycle^45^. Formation of clones with different sizes may be a general phenomenon for the genes that are expressed unevenly and are required for cell proliferation or cell survival. Varied levels of *sona* and *vg* expression in different parts of wing discs may be required to create regional differences in growth rate in order to form wings with proper size and shape.

What are the distinct functions of *sona*-expressing cells? In normal conditions, *sona* is expressed in a widely varing level  in a patchy pattern, and acts as a signal to promote cell proliferation for wing development. In irradiated conditions, the transcriptional level of *sona* dramatically increased among *sona-*expressing cells that may be required to cope against extensive cell death. This irradiation generated two major cell groups in the wing pouch. The first group of cells are determined to grow and proliferate or, alternatively, to die upon a high level of irradiation. The second group of cells are resistant to irradiation and send out signals to regulate the first group of cells. The first group of cells should express proteins for cell growth, cell proliferation, cell survival and cell death including Diap1and Rpr. The second group of cells should have capacity to sense the changes in environment and send out signals to regulate the first group of cells. In this study, we identified Sona as one of the proteins expressed in the second group of cells in a patchy pattern. Interestingly, a phosphorylated form of ribosomal protein S6 (pS6) that should be regulated in the first group of cells is also present in a patchy pattern^46,47^. We speculate that pS6 and *sona* may express in the first and the second groups of cells, respectively.

We showed that Sona induces Cyc D in nearby cells, which is consistent with the finding that overexpressed Sona increases tissue size (Fig. 7). Cyc D is known as a target protein of Wnt signaling in mammals^40–43^, and acts in a G1 phase of cell cycle that responds to mitogens^48^. In flies, overexpression of Cyc D/Cdk4 accelerates cell division in proliferating wing disc cells while increasing the cell size in post-mitotic cells by endoreplication^49^. Because Cyc D is required for cell division of surviving cells, it should be upregulated in Diap1-expressing cells. Indeed, *Diap1* is induced by Yorki and STAT in Hippo pathway and JAK-STAT pathway, respectively, and these two pathways are essential for cell proliferation in a cell autonomous manner^25,26^. An important question is the identity of the direct signal(s) that upregulate Cyc D. Secreted Sona may act as a direct signal or, as a metalloprotease, may modulate the activity of other signaling molecules such as Wg. It has been reported that irradiation activates a damage-activated enhancer BRV118 of the *wg* gene^50,51^. Therefore, irradiation changes the transcription pattern of both *sona* and *wg*, and may coordinate the functions of Sona and Wg for induction of Cyc D, although Sona may activate yet another signaling molecules.

An interesting new mechanism to repair tissue damage has been recently discovered, in which cells of the presumptive hinge region in the wing disc migrate into the pouch region after irradiation^52^. These hinge cells also migrate into the pouch region when the pouch cells are killed by *hid* expression^53^. These hinge cells are resistant to cell death due to the activation of Wg signaling and STAT signaling that suppress the transcription of *rpr*^52^. Resistance to irradiation and lack of *rpr* expression are also characteristics of *sona*-expressing cells, suggesting that the *sona*-expressing cells in the wing pouch may be functionally related to the radiation-resistant hinge cells. Because intense irradiation physically induces DNA breakage even in these radiation-resistant cells, the mechanism by which these *sona*-expressing cells cope against DNA damage is an important question to be explored.

Several mammalian metalloproteases in ECM are also shown to prevent apoptosis. Overexpression of MMP-15 (matrix metalloprotease-15) prevents apoptosis of Hela and human adenocarcinoma^54^. Furthermore, ADAM-12 increases apoptosis of stromal cell but decreases that of tumor cells^55^. In ADAMTS family, ADAMTS20 is shown to be required for cell survival. ADAMTS20 is mutated in *belted* (*bt*) mice that show cell death of melanoblast^56^. Sona is also involved in cell survival by increasing resistance against irradiation and by promoting cell proliferation of neighboring cells. Identification of Sona substrate and its function will greatly help understand the role of Sona in cell survival, which is currently ongoing in our laboratory.

## Materials and Methods

### *Drosophila* strains

All *UAS-sona RNAi* lines and *sona* mutants generated in our lab and characterization of *sona-Gal4* line (P{GawB}CG9850) are described^28^. *P{lacW}Diap1*^*j5C8*^*/TM3* was used as *Diap1-lacZ* line^57,58^. *UAS-Diap1*, *UAS-lacZ*, *rpr-lacZ*, *wg-lacZ*, *UAS-GFP*, *UAS-p35*, *hsFLP*; *FRT42D ubiGFP*, *FRT42D ubi-GFP*, *ptc-Gal4*, *en-Gal4*, *tubulin-Gal4* and *apterous-Gal4* were obtained from Bloomington stock center. *UAS-rpr RNAi* was obtained from VDRC (101234). *ci-Gal4* is described in^59^. Fly cultures were carried out at 25 °C unless otherwise indicated.

### Adult wing mounting

Wing of 2–7 days old adult flies were dissected and mounted in Gary’s Magic Mountant (Mixture of Canada Balsam and methyl salicylate, 4:1).

### Generation of mitotic clones and RNAi clones

Mitotic clones were generated by FLP-mediated recombination^60^. *sona*^*13*^ *FRT42D/CyO* or *sona*^*47*^ *FRT42D/CyO* flies were crossed to *hsFLP*; *FRT42D ubi-GFP* lines^28^. Clones were induced by heat shock at 37 °C for 1 hour during first or second instar larval stages, and late 3^rd^ instar larvae were dissected. For the generation of *sona* *RNAi* clones, *hsFLP* > *Actin* > *CD2* > *Gal4* flies^61^ were crossed with *UAS-GFP*; *UAS-sona RNAi-1*^*11–4*^ line and their progeny were heat-shocked for one hour at 37 °C to induce the clones, and dissected when they were late 3^rd^ instar larvae. For control clones, *UAS-GFP* line was used.

### Immunocytochemistry

Wing discs from wandering third-instar larvae were dissected and processed as described^62^. DAPI staining solution (Boehringer Mannheim) was used in the first washing step after secondary antibody staining. Discs were mounted in Vectashield (Vector Laboratories). The images were acquired using Zeiss LSM laser scanning confocal microscope and presented using Adobe Photoshop.

Primary antibodies were used in the following dilutions: sheep anti-GFP (Abd serotec), 1:100; chicken anti-*β*-gal (ab 9361), 1:100; rabbit anti-cleaved Caspase-3 (Cell Signaling Technology), 1:250; rabbit anti-cleaved Dcp-1 (Cell Signaling), 1:100; rabbit anti-Sona-Pro^28^, 1:500; mouse anti-Diap1 (a gift from Dr. Soon Ji Yoo^21^,) 1:100; mouse anti-Cyc D (Santa Cruz), 1:200; rabbit anti-Dlg^63^, 1:500; rabbit anti-PH3 (Millipore), 1:200; rat anti-HA (Roche), 1:150. Secondary antibodies were from Jackson Immunoresearches.

### Apoptosis induction by irradiation

Flies were grown at 25 °C until their progeny reached second to third instar larvae, and were treated with the indicated doses of γ–radiation (1,500 or 4,500 rad). They were allowed to grow for 24 hours after irradiation and dissected at the wandering 3^rd^ instar larvae.

### BrdU feeding

The protocol has been modified based on several reports^64–66^. Embryos of *sona* > *lacZ* genotype were cultured at 25 °C until they reached to the 1^st^ or 2^nd^ instar larvae. Right after irradiation at 4,500 rad, 0.5 mg/ml BrdU were poured evenly on top of the food containing irradiated larvae. After 24 hours of BrdU feeding, 3^rd^ instar larvae were dissected in Schneider’s insect culture medium (M3 medium). For control experiments, unirradiated 1^st^ and 2^nd^ instar larvae were fed in BrdU containing food for 24 hours. The discs were fixed for 20 min in PBTx (phosphate buffered saline, 0.3% Triton X-100) containing 5% paraformaldehyde.

### Image acquisitions and analysis

To quantify the degree of colocalization, the confocal images from two channels were compared using coloc2 analysis package in Fiji software^67^. To minimize the background signal, the threshold values were automatically set according to the Cotes method. For testing Cotes’ significance, the point spread function (PSF) was set to 50 with the number of iteration of 10. Mander’s Colocalization Coefficient (MCC) was presented within results. The representative 2D histograms were also generated using Fiji software.

## Supplementary information

Supplementary Information
