# Author Correction: An ADAMTS Sol narae is required for cell survival in *Drosophila*

**DOI:** 10.1038/s41598-019-53741-x

**Published:** 2019-11-14

**Authors:** Orkhon Tsogtbaatar, Jong-Hoon Won, Go-Woon Kim, Jeong-Hoon Han, Young-Kyung Bae, Kyung-Ok Cho

**Affiliations:** 1 Department of Biological Sciences, Korea Advanced Institute of Science and Technology, 291 Daehak-ro, Yuseong-gu, Daejeon, Korea; 2 Present Address: Center for Bioanalysis, Korea Research Institute of Standards and Science, 267 Gajung-ro, Yuseung-gu, Daejeon, Korea

Correction to: *Scientific Reports* 10.1038/s41598-018-37557-9, published online 04 February 2019

This Article contains an error in the Acknowledgements section.

“We are grateful to K.-W. Choi, S.-T. Hong and colleagues in our lab for discussion and comments on manuscript. We also thank to D.-G. Cho for irradiating flies, and O.-K. Lee and B.-S. Kim for technical assistance and fly maintenance. We thank S. J. Yoo for Diap1 antibody and J.-K. Chung for *Diap1-lacZ* fly strain. We acknowledge Bloomington Stock Center, Drosophila Genetic Resource Center and Developmental Studies Hybridoma Bank for fly strains and antibodies. This research was supported by National Research Foundation R1A1A301573 and N01170193, and National Research Council of Science and Technology Grant DRC-14-2-KRISS”.

should read:

“We are grateful to K.-W. Choi, S.-T. Hong and colleagues in our lab for discussion and comments on manuscript. We also thank to D.-G. Cho for irradiating flies, and O.-K. Lee and B.-S. Kim for technical assistance and fly maintenance. We thank S. J. Yoo for Diap1 antibody and J.-K. Chung for *Diap1-lacZ* fly strain. We acknowledge Bloomington Stock Center, Drosophila Genetic Resource Center and Developmental Studies Hybridoma Bank for fly strains and antibodies. This research was supported by National Research Foundation R1A1A301573 and 2017R1A2B4009254, and National Research Council of Science and Technology Grant DRC-14-2-KRISS”.

