# Supplementary material for: An ADAMTS Sol narae is required for cell survival in Drosophila
Source: Sci Rep. 2019 Feb 4;9:1270. doi: 10.1038/s41598-018-37557-9 (PMC6362049; doi:10.1038/s41598-018-37557-9)

An ADAMTS Sol narae is required for cell survival in *Drosophila*

Orkhon Tsogtbaatar, Jong-Hoon Won, Go-Woon Kim, Jeong-Hoon Han, Young-Kyung Bae † * and Kyung-Ok Cho*

Supplementary Information

Supplementary Figure S1. *sona RNAi*-induced cell death.

(**a, b**) Expression of *sona RNAi-1*$^{3\text{-}23}$ by *en-Gal4* driver results in reduced wing size (**b**), especially in the posterior region compared to the control *en>Gal4/+* wing (**a**). (**c**) CC3 was detected (**c'**) and the number of nuclei (**c"**) was less in the posterior region of *en>sona-RNAi*$^{3\text{-}23}$. (**d, e**) The basal region of *ap>sona RNAi-1*$^{11\text{-}4}$ wing disc exhibiting cell death and pyknotic nuclei (arrowheads in **e'**,**e"**) in comparison to control *ap>GFP* wing disc (**d-d"**).

Supplementary Figure S2. Correlation between the pattern of GFP and Sona protein in *sona>GFP* discs.

(**a**) Sona-Pro antibody and GFP in *sona>GFP* disc are visualized to show the correlation between Sona protein and GFP driven by *sona-Gal4* driver. (**b**) The boxed region in (**a**) is magnified.

Supplementary Figure S3. Comparison between the control clones and *sona*^{13} clones.

Control and *sona*^{13} clones are generated in the first to second instar stage.

Supplementary Figure S4. *sona RNAi* clones are smaller than control clones.

Control and *sona RNAi-1*$^{11\text{-}4}$ clones were generated at different developmental time points as indicated in (**a**). An example of the control and RNAi clones are shown in (**b-e**) and (**f-i**), respectively, the average size of clones were plotted in (**j**). Small clones were generated when the clones were generated in the time point V. Compared to wild-type clones (**k**) and *sona RNAi* clones (**l,m**) are absent in the regions marked with arrows.

Supplementary Figure S5. Relationship between Sona and CC3 signals in the wing disc.

(**a**) A line was drawn in the pouch area of *sona>GFP* wing disc, and the intensities of GFP (representing *sona* transcription) and CC3 were measured using Image J. (**b,c**) Confocal images of 10 wing discs were analyzed by co-localization tests using FIJI. The averaged value for each correlation value and the standard errors were shown.

Supplementary Figure S6. Mander's coefficients and 2D histogram of controls.

(**a**) *en>GFP* discs stained for GFP and Dcp. Anti-Dcp Antibody was used instead of anti-CC3 antibody because Dcp was more easily detected than CC3. (**b,c**) Mander's coefficient and 2D histograms are shown in **b** and **c**, respectively. Mander's tM1 value, 0.793, is overestimated because all DCP signal in the posterior region is also GFP-positive. (**d**,**e**) The same *sona>GFP* image with two different colors (**d**) were used to generate the 2D histogram (**e**).

Supplementary Figure S7. Expressions of *sona* and Diap1 show negative correlation.

(**a,b**) Confocal images of 13 (before irradiation) and 24 (24 hours after 4,500 rad irradiation) wing discs were analyzed as described in Supplementary Fig. S5.

Supplementary Figure S8. Expression pattern of GFP driven by *tubulin-Gal4* before and after irradiation.

(**a,b**) Unirradiated (**a**) and irradiated (**b**) *tubulin >GFP* disc fixed 24 hours after 4,500 rad shows no change in the pattern of GFP.

Supplementary Figure S9. Origin of *sona*$^{+}$ clusters revealed by 3D image analysis.

(**a,b**) Combined images of 44 (**a**) and 50 (**b**) z-sectioned images of unirradiated and irradiated *sona>GFP* discs, **a'** and **b'** images are GFP only. **a"** and **b"** images are a single apical image from z-sectioned image from **a** and **b**. (**c,d)** The magnified image from the region in **b** marked with an asterisk. Arrowheads show that the *sona*$^{+}$ cluster is indeed a cell with nucleus. Arrows in **c'** that is generated from the 3D image show continuous *sona*$^{+}$ cell groups. **d** is a tilted image generated from the same 3D image.

Supplementary Figure S10. The level of *sona* transcription is increased 24 hours after irradiation.

sona>GFP larvae were irradiated and wing discs were dissected after 24 hours. The level of GFP measured in 11 unirradiated *sona>GFP* discs and 33 irradiated *sona>GFP* discs by Image J were plotted.

Supplementary Figure S11. The level of Sona protein is increased in the cells with higher level of *sona* transcription in irradiated *sona>GFP* discs.

(**a**) Wing discs from irradiated *sona>GFP* larvae stained for GFP and Sona. *sona*$^{+}$ clusters detected by GFP are clearly seen in **a'**, and Sona protein is visualized with Sona-pro antibody in **a"**. (**b**) A 2.5D image was generated from the image in **a** using Zen

program. Arrows indicate the regions with high levels of both GFP and Sona, and asterisks indicate the regions with low levels of both GFP and Sona.

Supplementary Figure S12. Transient expression of Sona promotes development in irradiated flies.

sona-Gal4/+; Gal80^{ts}/+ control flies and *sona-Gal4/+; Gal80^{ts} UAS-sona/+* experimental flies at 18°C for 6 days until the mid to late third instar stage, and then cultured them at 29°C for six hours to transiently induce Sona expression. Flies were then irradiated with 3,000 rad or 4,500 rad, cultured at 18°C, and measured the numbers of larvae and pupae on the wall of culture tube. The horizontal axis starts with the first day the larvae appeared as 1 and followed by 2nd to 18th days. The vertical axis shows the number of larvae and pupae.

Supplementary Figure S13. Sona induces Cyc D in neighboring cells.

(**a,b**) *ci> GFP; Gal80^{ts}* (**a**) and *ci>GFP, sona, Gal80^{ts}* (**b**) larvae were cultured for 10 hours at 29°C. Cyc D was expressed in vicinity of Sona-expressing cells (**b**, arrow) but not in control discs (**a**).

Supplementary Figures

Supplementary Figure S1

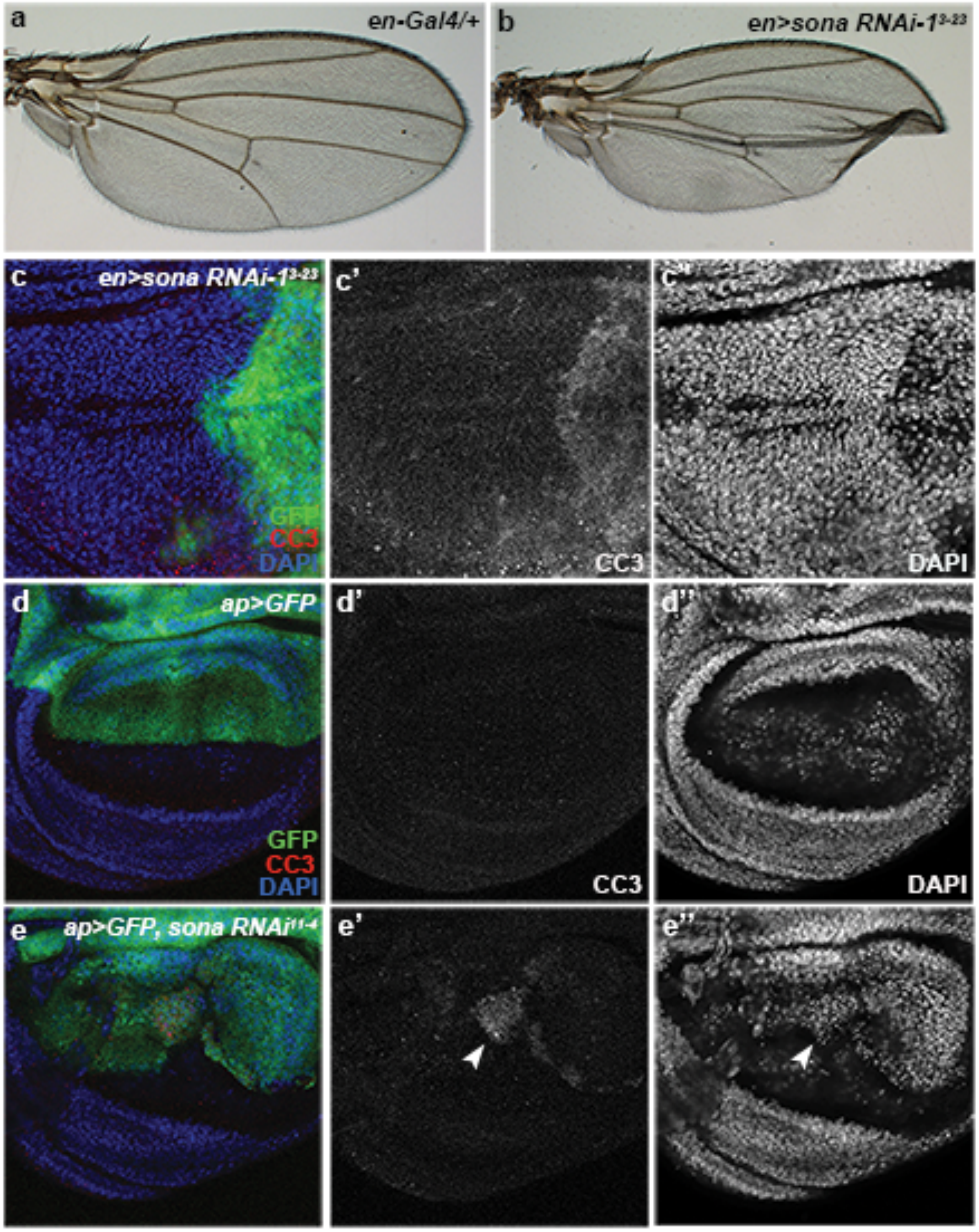

Supplementary Figure S2

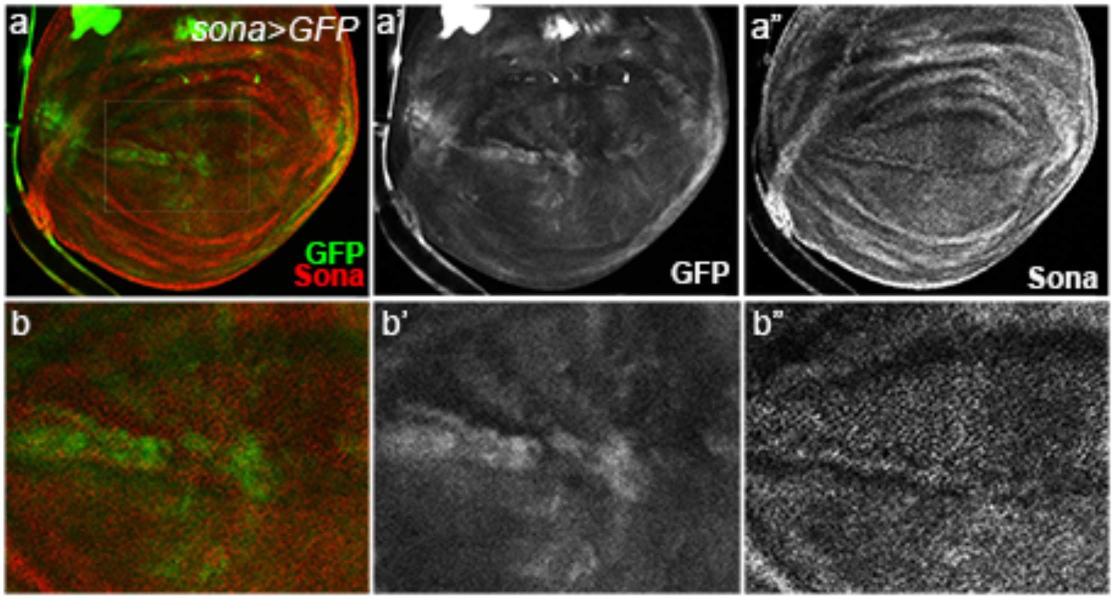

Supplementary Figure S3

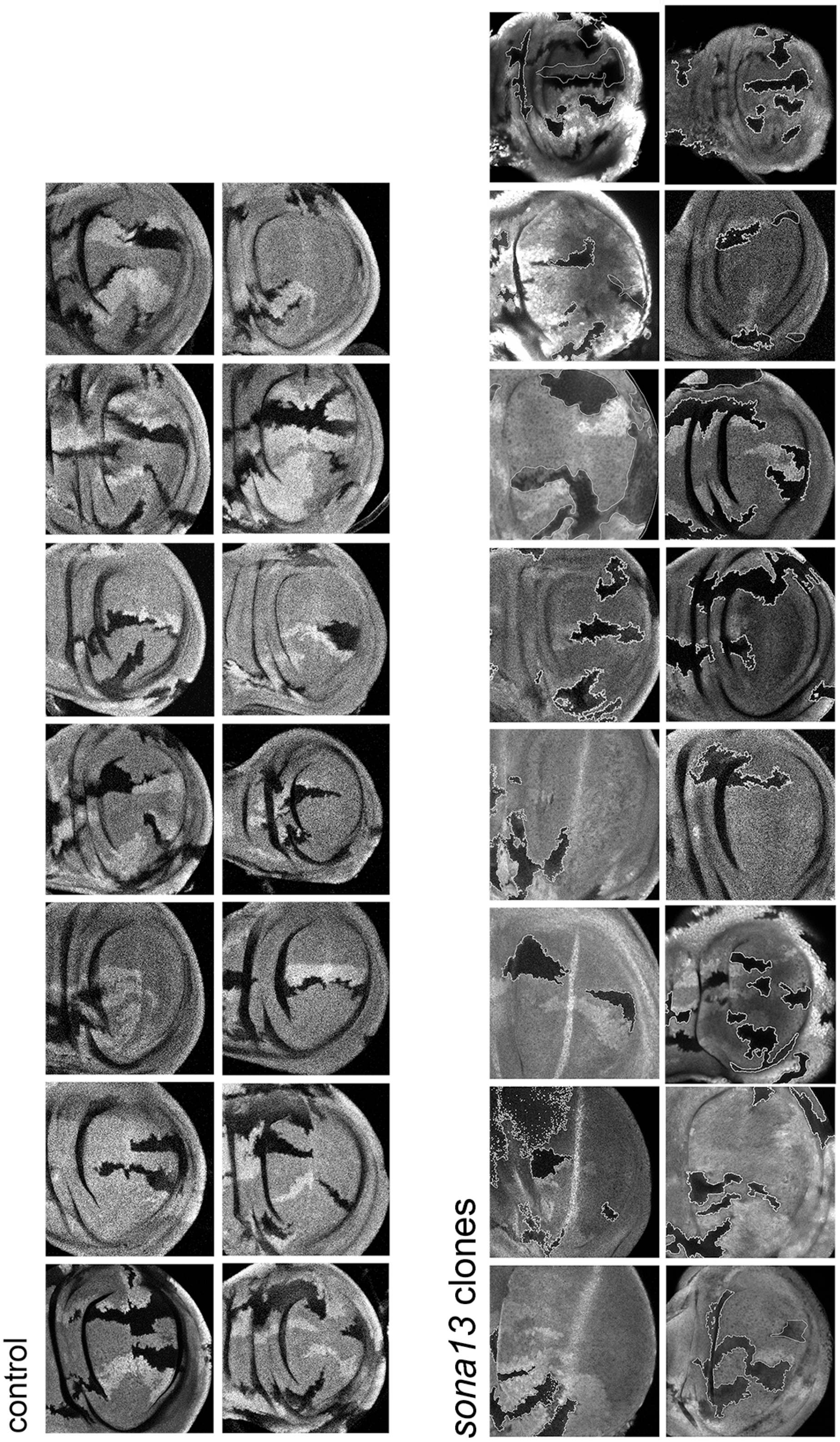

Supplementary Figure S4

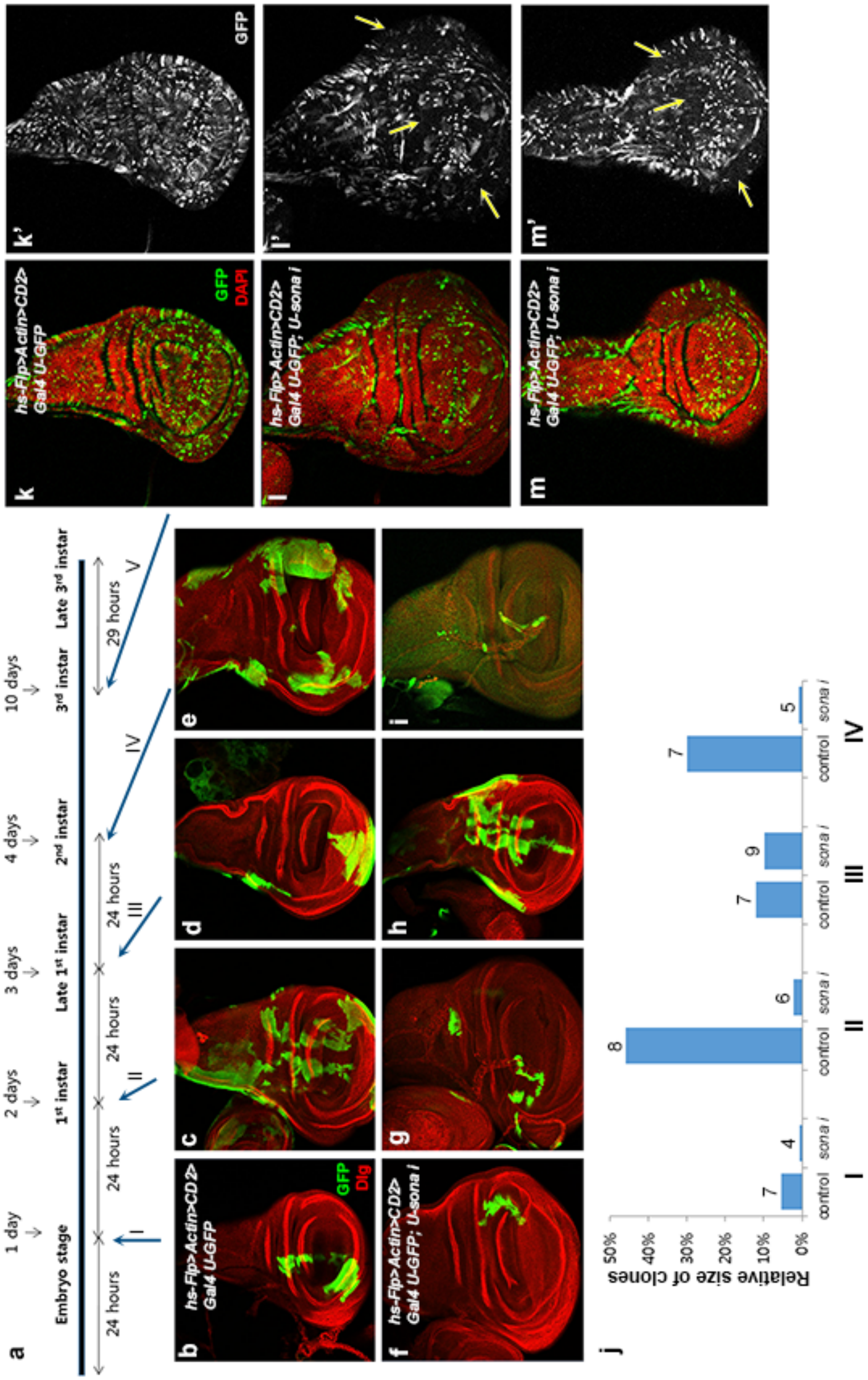

Supplementary Figure S5

a

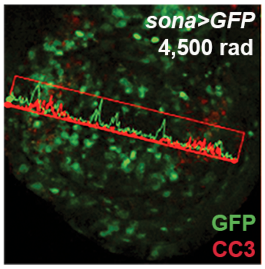

b

	24 hr 4,500 rad Average ± S.E. (n=10)
**Manders' tM1 (fraction of Sona+ when Cas3 is present)**	0.13±0.05
**Manders' tM2 (fraction of Cas3+ when Sona is present)**	0.15± 0.06

c

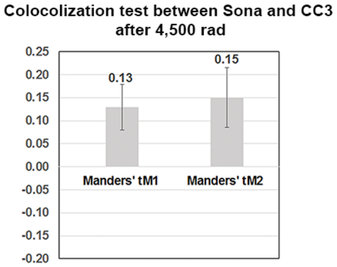

Supplementary Figure S6

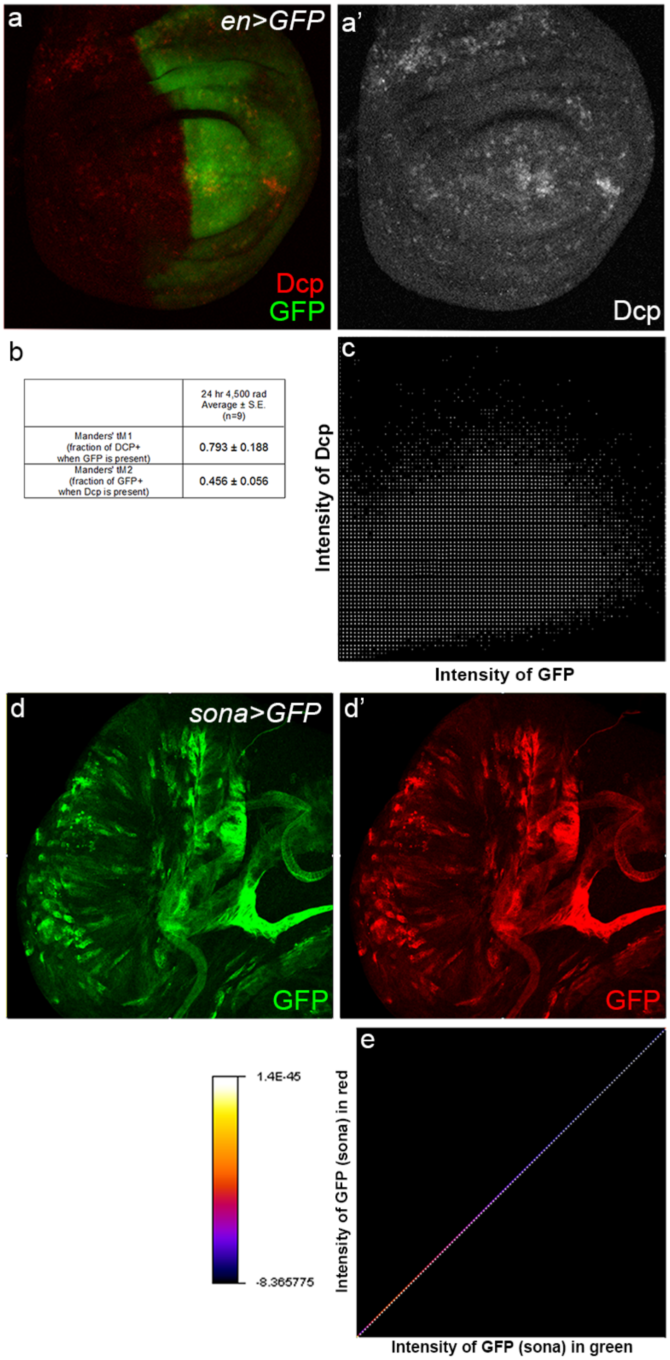

	24 hr 4,500 rad Average ± S.E. (n=9)
Manders' tM1 (fraction of DCP+ when GFP is present)	0.793 ± 0.188
Manders' tM2 (fraction of GFP+ when Dcp is present)	0.456 ± 0.056

Supplementary Figure S7

a

	BEFORE (n = 13)	24 hr 4500 rad (n=24)
Manders' tM1<br><br>(fraction of Sona+ when Diap1 is present)	0.25 ± 0.05	0.23 ± 0.02
Manders' tM2<br><br>(fraction of Diap1+ when Sona is present)	0.31 ± 0.04	0.22 ± 0.02

b

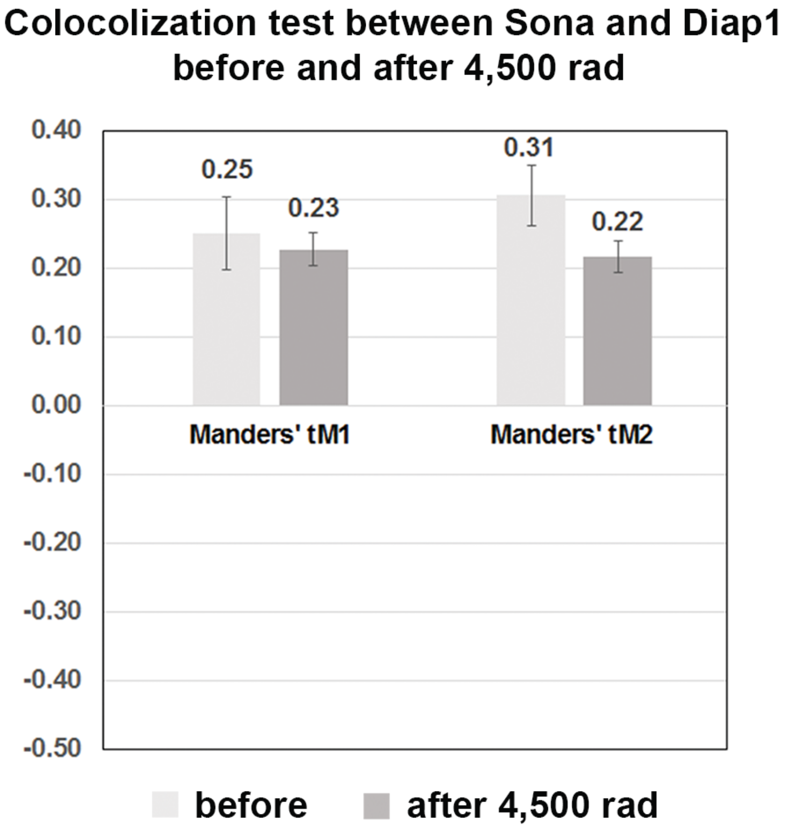

Supplementary Figure S8

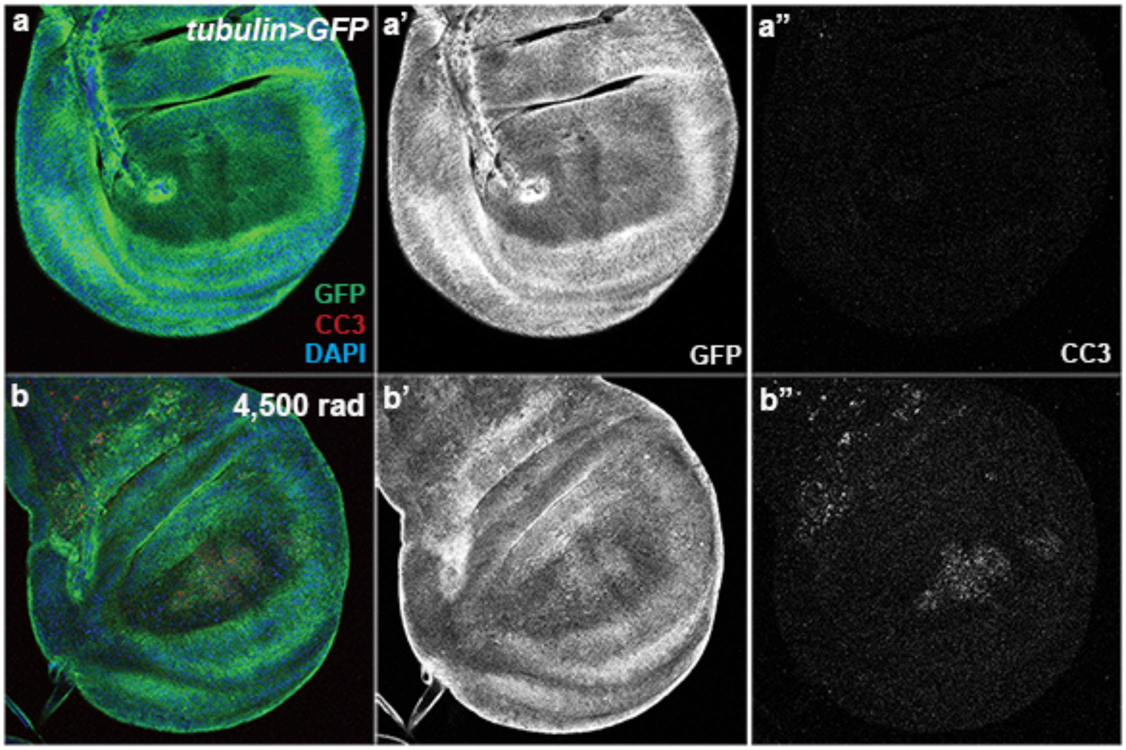

Supplementary Figure S9

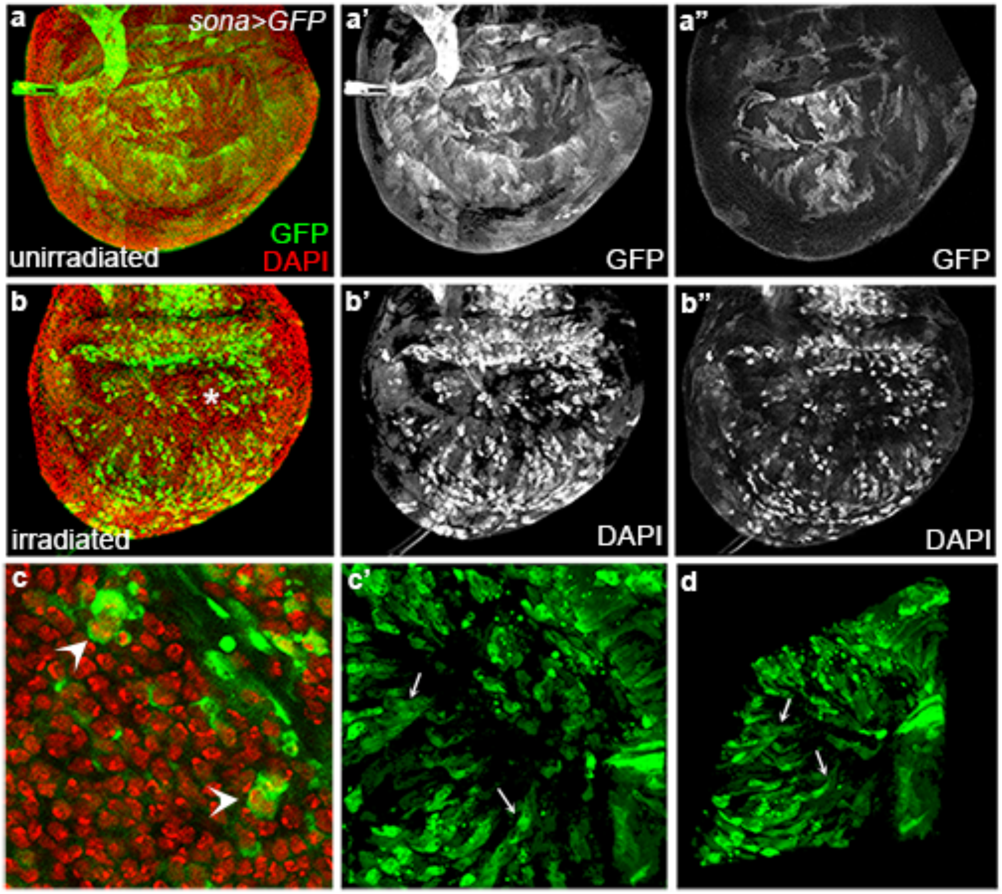

Supplementary Figure S10

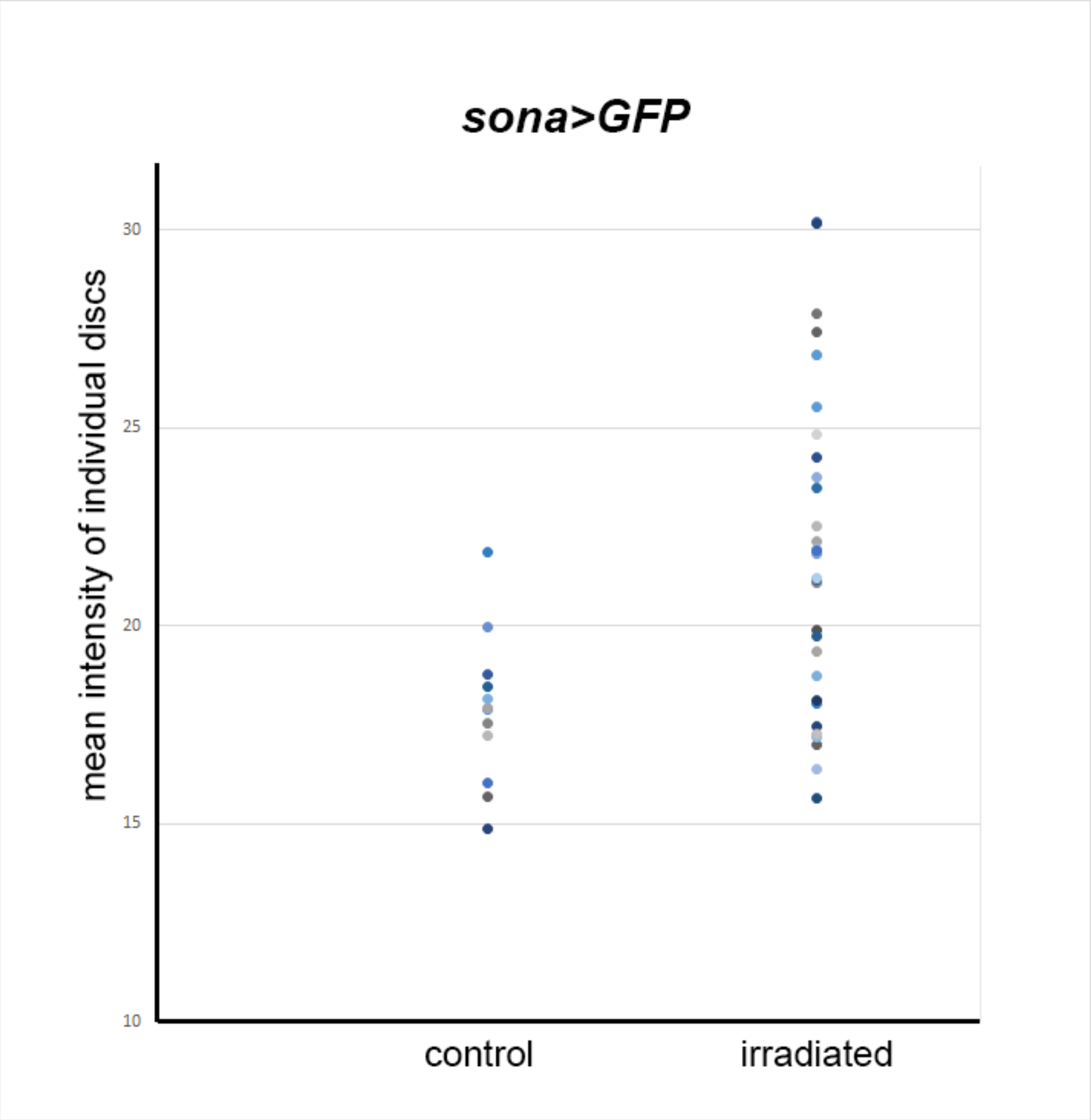

Supplementary Figure S11

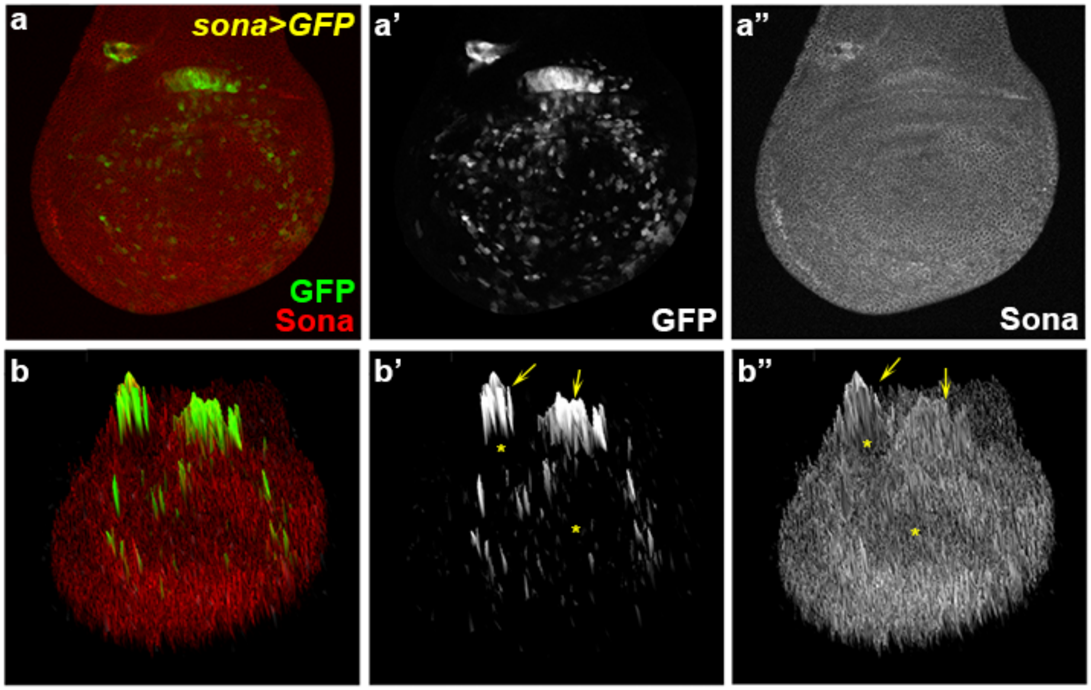

Supplementary Figure S12

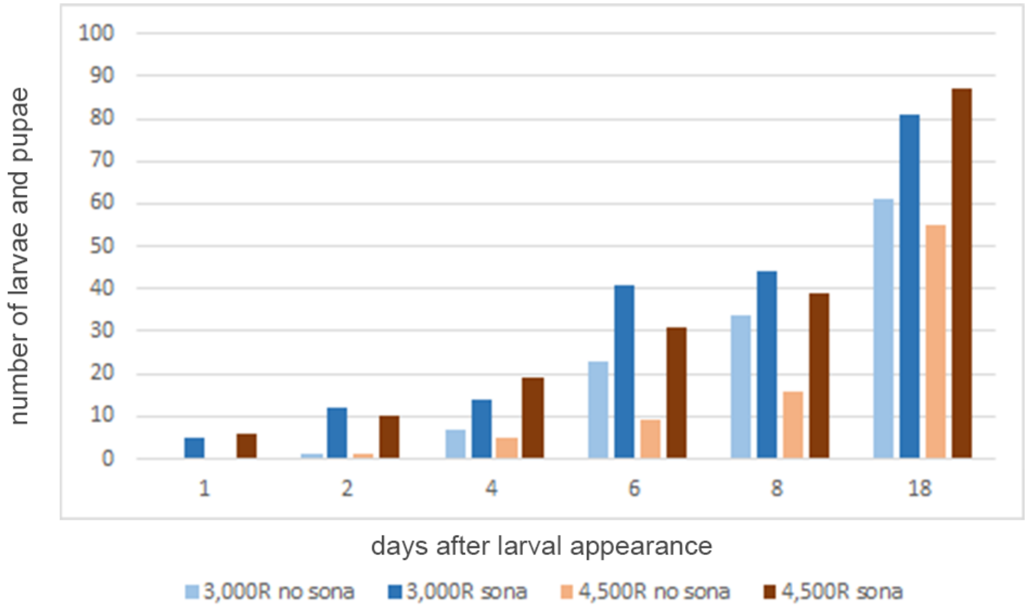

Supplementary Figure S13

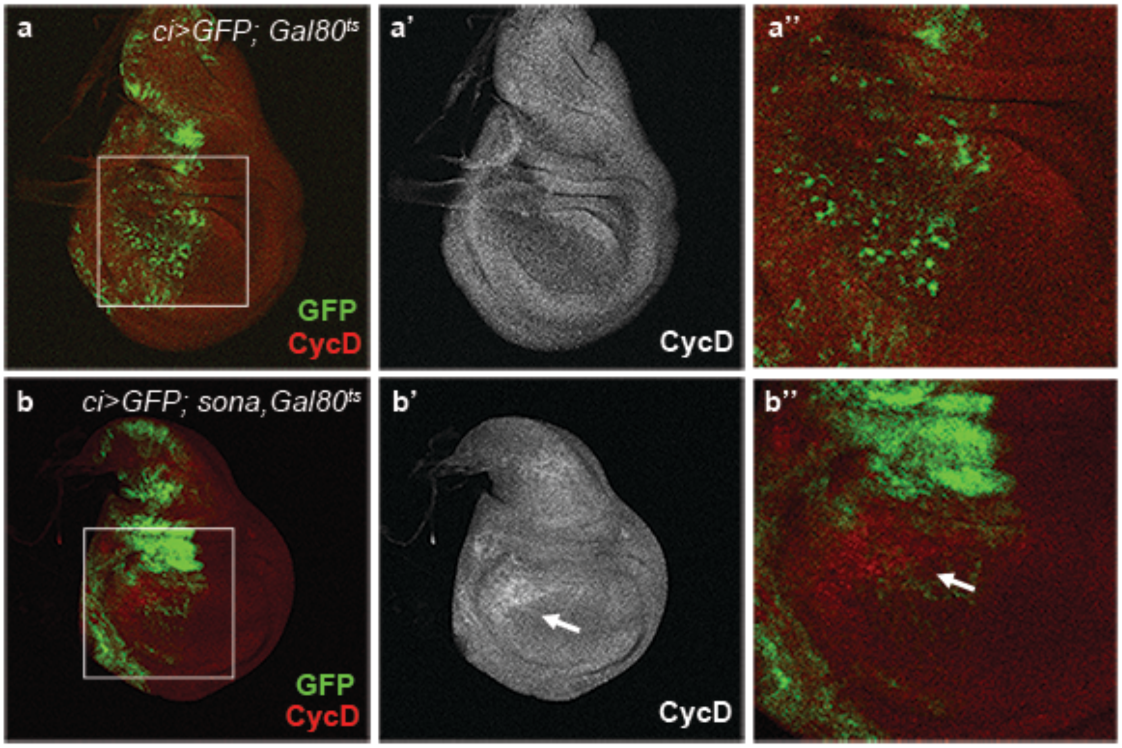

Supplement: Supplementary file 1 — Supplementary Information [file 41598_2018_37557_MOESM1_ESM.pdf]
